# Visible‐Light‐Activated Molecular Machines Kill Fungi by Necrosis Following Mitochondrial Dysfunction and Calcium Overload

**DOI:** 10.1002/advs.202205781

**Published:** 2023-01-30

**Authors:** Ana L. Santos, Jacob L. Beckham, Dongdong Liu, Gang Li, Alexis van Venrooy, Antonio Oliver, George P. Tegos, James M. Tour

**Affiliations:** ^1^ Department of Chemistry Rice University Houston TX 77005 USA; ^2^ IdISBA – Fundación de Investigación Sanitaria de las Islas Baleares Palma 07120 Spain; ^3^ Servicio de Microbiologia Hospital Universitari Son Espases Palma 07120 Spain; ^4^ Office of Research Reading Hospital Tower Health, 420 S. Fifth Avenue West Reading PA 19611 USA; ^5^ Smalley‐Curl Institute Rice University Houston TX 77005 USA; ^6^ Department of Materials Science and NanoEngineering Rice University Houston TX 77005 USA; ^7^ NanoCarbon Center and the Welch Institute for Advanced Materials Rice University Houston TX 77005 USA

**Keywords:** antifungal, fungal mitochondrial phospholipids, molecular machines, reduction of infection‐associated mortality and fungal burden, visible light activation

## Abstract

Invasive fungal infections are a growing public health threat. As fungi become increasingly resistant to existing drugs, new antifungals are urgently needed. Here, it is reported that 405‐nm‐visible‐light‐activated synthetic molecular machines (MMs) eliminate planktonic and biofilm fungal populations more effectively than conventional antifungals without resistance development. Mechanism‐of‐action studies show that MMs bind to fungal mitochondrial phospholipids. Upon visible light activation, rapid unidirectional drilling of MMs at ≈3 million cycles per second (MHz) results in mitochondrial dysfunction, calcium overload, and ultimately necrosis. Besides their direct antifungal effect, MMs synergize with conventional antifungals by impairing the activity of energy‐dependent efflux pumps. Finally, MMs potentiate standard antifungals both in vivo and in an ex vivo porcine model of onychomycosis, reducing the fungal burden associated with infection.

## Graphical abstract

Molecular machines (MMs) drilling at millions of rotations per second upon light activation eliminate planktonic and biofilm fungal populations without inducing resistance development. The antifungal effect results from MM binding to fungal mitochondrial phospholipids, leading to mitochondrial dysfunction, calcium overload, and necrosis. Besides their direct action, MMs enhance the activity of conventional antifungals in vitro, in vivo, and ex vivo.

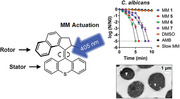

## 1 Introduction

Every year, over 1.6 million people die from fungal infections worldwide,^[^ ^1^ ^]^ with an estimated cost of $7.2 billion in the United States (US) alone.^[^ ^2^ ^]^ Fungi are typically opportunistic pathogens that exploit vulnerabilities in their host's weakened immune system. Therefore, the increased prevalence of fungal infections may be partially attributable to medical advances that have improved the survival rates of otherwise critically ill individuals such as patients with cancer or acquired immunodeficiency syndrome (AIDS) and transplant recipients.^[^ ^3^ ^]^

The treatment of fungal infections is challenged by the fact that there are only three major classes of antifungals in clinical use: azoles, echinocandins, and polyenes. Moreover, despite their efficacy, existing antifungal drugs are limited by toxicity, drug interactions, and low bioavailability.^[^ ^4^ ^]^ Fungal infection treatment is further complicated by increased antifungal resistance associated with widespread therapeutic and prophylactic antifungal use, which challenges the resilience of our current antifungal armamentarium.^[^ ^5^ ^]^

Although antifungal resistance continues to increase, the development of new antifungal agents has been slow. Indeed, the modern era of antifungal drug development has mostly been characterized by incremental changes to existing drugs that act on two main fungal targets: the cell membrane and the cell wall.^[^ ^6^ ^]^ The scarcity of fungal‐specific targets is problematic because antifungal cross‐resistance is widespread.^[^ ^7^ ^]^ Alarmingly, fungal strains that are resistant to all classes of commonly prescribed antifungals, that is, pan‐resistant, and for which there is currently no effective treatment, are becoming increasingly frequent.^[^ ^8^ ^]^

The Coronavirus disease 2019 (COVID‐19) pandemic has only exacerbated the problem of antimicrobial resistance, including antifungal resistance. COVID‐19 has been associated with an increased risk of fungal infections, including infections resistant to antifungal treatment, and fungal co‐infections have been found to contribute to COVID‐19‐associated mortality.^[^ ^9^ ^]^ Climate change^[^ ^10^ ^]^ and a growing number of vulnerable people due to age and/or underlying diseases, such as diabetes,^[^ ^11^ ^]^ are expected to further aggravate the antifungal resistance crisis in the coming years. Therefore, the identification of antifungal therapies with new targets and/or mechanisms of action that are not susceptible to the rapid development of resistance is now more important than ever to combat antifungal resistance and preserve the viability of existing antifungal agents.

**Figure 1:**
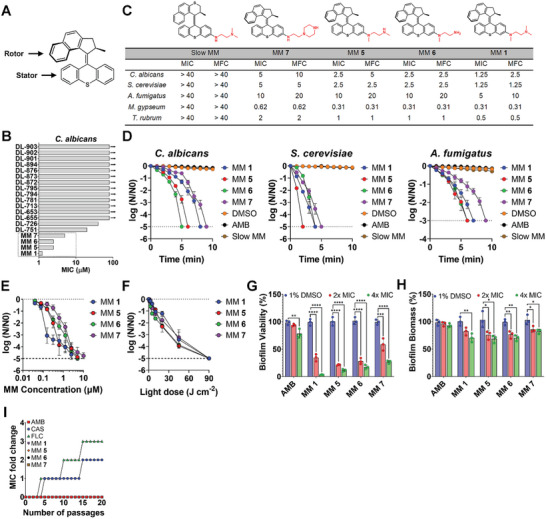
MMs show antifungal activity against planktonic cells and established biofilms. A) General structure of an MM. MMs consist of a stator and a rotor that is light‐activated. After light activation, the rotor portion of the molecule undergoes successive cycles of unidirectional rotation around the central carbon–carbon double bond, resulting in a fast (≈3 MHz) or slow (≈0.1 Hz) drill‐like motion, depending on the molecular design. B) Minimum inhibitory concentration (MIC, µm) of the different MMs investigated in this study in *Candida albicans* in the presence of 405‐nm light (87.6 J cm^−2^). The chemical structures of all compounds tested are shown in Table S1, Supporting Information. C) Chemical structures of the most potent antifungal MMs identified in this study, their MIC, and minimal fungicidal concentration (MFC) in different fungal strains. Results are shown as the average of at least three biological replicas. Concentration is expressed in µm. D) Time‐kill curves of different fungal strains treated with visible‐light‐activated MMs (2× MIC) or 1% DMSO in the presence of 405‐nm light at 292 mW cm^−2^ or control antifungal amphotericin B (AMB, 4× MIC). E) Concentration‐dependent killing of *C. albicans* by different MMs in the presence of 405‐nm light (87.6 J cm^−2^). F) Light dose‐dependent killing of *C. albicans* by different MMs at 2× MIC. The killing was assessed as the reduction in colony‐forming units (CFU) expressed as the logarithm of base 10 of the ratio between the CFU at each time point (*N*) and the CFU at time zero (*N*0). The results are expressed as the average of at least three replicates ± the standard error of the mean. The dashed line denotes the limit of detection of the method. G) Reduction of *C. albicans* biofilm viability by amphotericin B (AMB), 1% DMSO, or different MMs (2×, 4× MIC) in the presence of 405‐nm light (5 min at 292 mW cm^−2^). H) Reduction of *C. albicans* biofilm biomass by amphotericin B (AMB), 1% DMSO or different MMs (2×, 4× MIC) in the presence of 405‐nm light (5 min at 292 mW cm^−2^). The results are the average of at least three independent replicates ± the standard deviation. Asterisks denote the significance of the differences in pairwise comparisons with 1% DMSO controls performed in GraphPad Prism. *p <0.05, **p <0.01, ***p <0.001, ****p <0.0001. I) Development of resistance to conventional antifungals (caspofungin, CAS, fluconazole, FLC, or amphotericin B, AMB) or different visible‐light‐activated MMs in *C. albicans*, assessed as the MIC fold change over 20 cycles of repeated treatment. Note that curves for amphotericin B (AMB), MM **1**, MM **5**, MM **6**, and MM **7** are superimposed. Unless otherwise indicated, the results for MMs and DMSO are always reported in the presence of light.

Synthetic nanomaterials that are not targeted by the natural defensive arsenal of microorganisms represent an unconventional approach to treating infections refractory to standard antimicrobials.^[^ ^12^, ^13^ ^]^ Molecular machines (MMs) (**Figure** **1**A) are examples of stimuli‐responsive compounds that, in response to light, undergo a sequential unidirectional conformational change, generating a drill‐like motion that can propel the molecule through lipid bilayers.^[^ ^14^ ^]^ These stimuli‐responsive systems are particularly promising because they enable antimicrobial attack using a mechanical mechanism at the molecular scale. MMs can be spatially and temporally activated by light, allowing precise localization and temporal control of antimicrobial action. If therapeutic effects can be achieved by mechanical rather than traditional chemical means, the selective pressure created by high antimicrobial doses can be reduced, retarding or mitigating the emergence of antimicrobial resistance.

Here, we describe the ability of 405‐nm‐visible‐light‐activated MMs to rapidly kill planktonic and biofilm fungi without resistance development via a new mechanism of action in which MMs bind fungal mitochondrial phospholipids, eliciting mitochondrial dysfunction, calcium overload, and necrosis following light activation. At sublethal concentrations, MMs also potentiated the effects of conventional antifungals, at least in part by impairing efflux pump function. Finally, MMs synergized with conventional antifungals in vivo, reducing mortality and fungal burden associated with systemic fungal infections, and ex vivo, outperforming monotherapy with conventional antifungals in reducing the fungal load in an onychomycosis porcine model.

## 2 Results

### 2.1 MMs Kill Planktonic and Biofilm Fungi without Resistance Development

19 fast, unidirectionally rotating (≈3 MHz) visible‐light‐activated MMs (Table S1, Supporting Information)^[^ ^15^ ^]^ and slow motor control (10^−6^ Hz) were examined for antifungal activity against a strain of the human pathogen *Candida albicans* isolated from a skin lesion (ATCC 18 804). Since substituted piperazines can improve molecule lipophilicity to increase antimicrobial activity,^[^ ^16^ ^]^ a piperazine‐modified molecular machine (MM **7**) was also investigated.

*C. albicans* cell suspensions were incubated with increasing concentrations of MMs and irradiated with 405‐nm light at 292 mW cm^−2^ for 5 min (87.6 J cm^−2^). The minimum inhibitory concentration (MIC) was defined as the MM concentration resulting in no visible fungal growth after irradiation with 87.6 J cm^−2^ of 405‐nm light.

The MICs of the different MMs for *C. albicans* varied from 1.25 to 80 µm (Figure 1B). The inhibitory effects of the most potent MMs (MM **1**, MM **5**, MM **6**, MM **7**), displaying MIC values ≤5 µm, were further investigated in the yeast *Saccharomyces cerevisiae* and the molds *Aspergillus fumigatus*, *Microsporum gypseum*, and *Trichophyton rubrum*. *S. cerevisiae* showed a susceptibility profile similar to that of *C. albicans*, with MIC values of 1.25–5 µm. Among molds, *A. fumigatus* had the highest mean MIC values (5–10 µm), whereas *M. gypseum* and *T. rubrum* were more sensitive to visible‐light‐activated MMs, with MIC values of 0.31–2.5 µm (Figure 1C).

The minimum fungicidal concentration (MFC), that is, the lowest MM concentration that killed ≥99.9% of the original inoculum,^[^ ^17^ ^]^ was similar to or at most twice the MIC (Figure 1C), demonstrating that MMs are indeed fungicidal and not just fungistatic.

The antifungal potential of the four most potent MMs was further investigated in time‐kill experiments by treating fungal strains with MMs (2 × MIC) or 1% dimethylsulfoxide (DMSO), followed by irradiation with 405‐nm light at 292 mW cm^−2^ for up to 10 min. A slow (≈10^−6^ Hz) MM control (Figure 1C), structurally homologous to MM **1** (≈3 MHz), was used to assess the importance of rotation speed for MM fungicidal activity. Amphotericin B (AMB, 4 × MIC, Table S2, Supporting Information) was used as a control antifungal.

MM treatment reduced *C. albicans* cell numbers to the limit of detection in 5 (MM **6**) to 9 min (MM **7**) (Figure 1D). In *S. cerevisiae*, population eradication was achieved in 2 (MM **5**) to 5 min (MM **7**) (Figure 1D). *A. fumigatus* cell number reduction to the limit of detection occurred from 6 (MM **5**) to 9 min (MM **7**) (Figure 1D). Non‐irradiated MMs and slow MMs had no significant effect on cell number (Figure 1D and Figure S1, Supporting Information), demonstrating the importance of light‐induced fast rotation rates for the fungicidal activity of MMs. Treatment with AMB resulted in a non‐significant reduction in cell numbers (Figure 1D). Under the same irradiation conditions, the killing of *C. albicans* by MMs varied in a concentration‐dependent manner (Figure 1E), with increasing MM concentrations enhancing killing. At the same MM concentration, the killing could also be remotely controlled by adjusting the light dose, with higher light doses leading to enhanced killing (Figure 1F).

The antibiofilm potential of the most effective visible‐light‐activated MMs (2 ×, 4 × MIC plus 87.6 J cm^−2^ of 405‐nm light) against mature *C. albicans* biofilms was evaluated in a 96‐well plate format using the 2,3‐Bis(2‐methoxy‐4‐nitro‐5‐sulfophenyl)‐2H‐tetrazolium‐5‐carboxanilide (XTT) assay^[^ ^18^ ^]^ and crystal violet assay^[^ ^19^ ^]^ to assess effects on viability and biomass, respectively, against the control antifungal AMB (2 ×, 4 × MIC). Compared with DMSO controls, visible‐light‐activated MMs reduced biofilm viability by up to 96% (MM **1**, *p* <0.0001), whereas AMB reduced biofilm viability by only 20% (*p* <0.01) (Figure 1G). Relative to DMSO controls, visible‐light‐activated MMs reduced biofilm biomass by up to 35% (MM **5**, *p* <0.05), whereas AMB treatment achieved only a non‐significant 6% reduction (Figure 1H).

Resistance development to visible‐light‐activated MMs was assessed by serial passage experiments. *C. albicans* cells surviving 0.5 × MIC of MM plus light (405‐nm at 87.6 J cm^−2^) were subjected to 20 cycles of repeated MM treatment. Unlike caspofungin (CAS) and fluconazole (FLC), repeated MM treatment did not increase the MM MIC (Figure 1I). Furthermore, antifungal‐resistant mutants did not exhibit cross‐resistance to MMs (Table S3, Supporting Information). A single‐step strategy to isolate MM‐resistant mutants was attempted, but no resistant colonies were recovered (Figure S2, Supporting Information).

### 2.2 Antifungal Mechanisms of MMs

The mechanisms of action of MMs were investigated using the human pathogen *C. albicans* under the same irradiation conditions (405‐nm light at 87.6 J cm^−2^) and varying MM concentrations (0.5 ×, 1 ×, or 2 × MIC) (Figure 1C). Comparison with 1% DMSO‐treated samples irradiated under similar conditions allowed discrimination between MM‐induced effects and those caused by irradiation alone.

**Figure 2:**
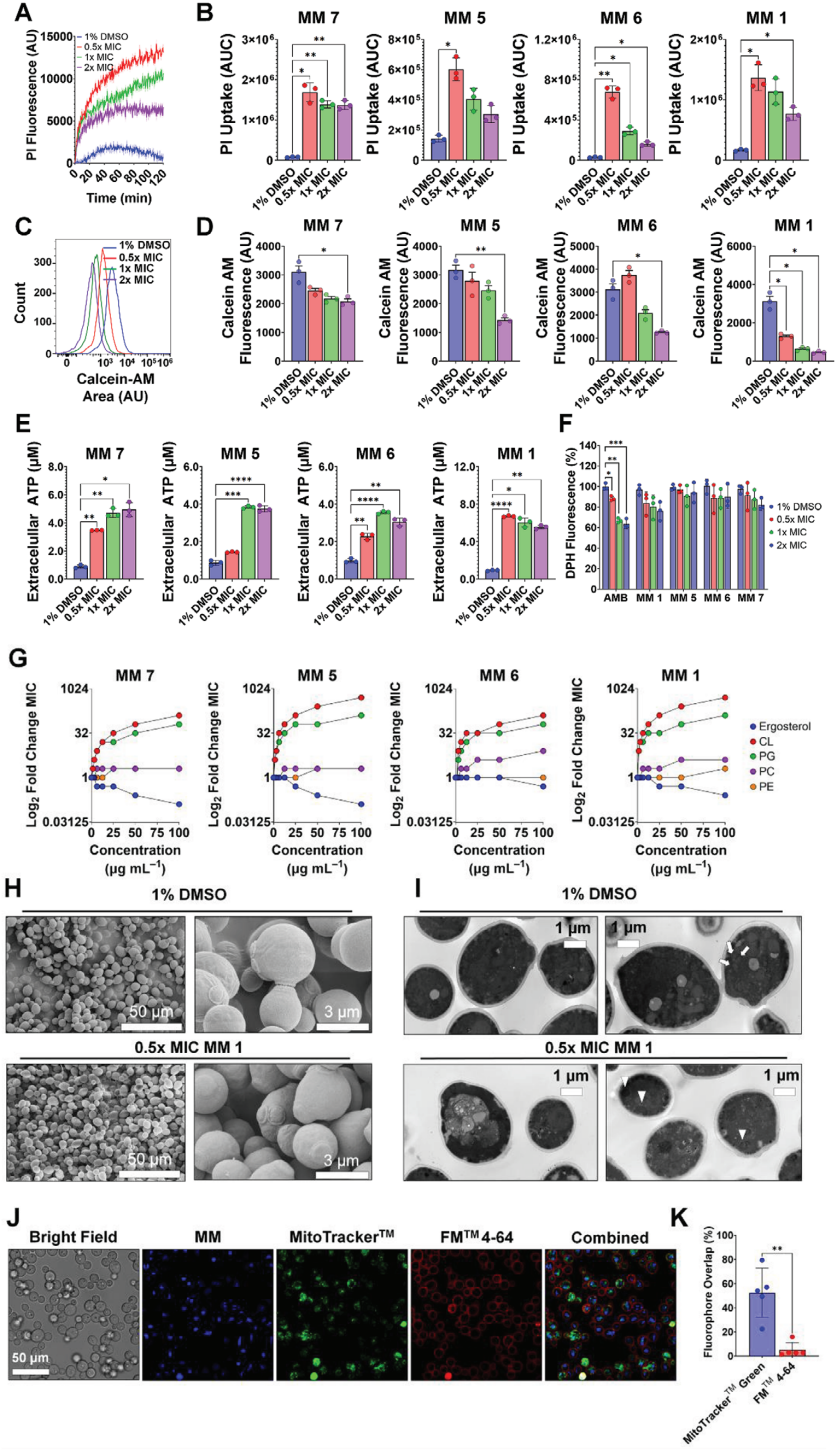
MMs bind fungal mitochondrial phospholipids. A) Representative temporal profile of PI fluorescence in *Candida albicans* treated with MM **1** (0.5–2 × MIC) or 1% DMSO and irradiated with 405‐nm light (87.6 J cm^−2^). Lines are the average of at least three biological replicates, and the shaded area is the standard error of the mean. B) PI uptake in *C. albicans* treated with different MMs (0.5–2× MIC) or 1% DMSO in the presence of 405‐nm light (87.6 J cm^−2^). PI uptake was calculated as the area under the curve (AUC) of the temporal profiles of PI fluorescence, as shown in (A). The results are the average of at least three independent replicates ± the standard deviation. C) Representative histogram of calcein AM fluorescence in *C. albicans* cells treated with 1% DMSO or MM **1** (0.5–2 × MIC) and irradiated with 405‐nm light (87.6 J cm^−2^), assessed by flow cytometry. D) Decrease in calcein AM fluorescence in *C. albicans* treated with 1% DMSO or different MMs (0.5–2× MIC) and irradiated with 405‐nm light (87.6 J cm^−2^). The results are expressed as the arithmetic mean ± the standard deviation of fluorescence obtained by flow cytometry. E) Extracellular ATP levels in *C. albicans* treated with increasing concentrations of different MMs (0.5–2× MIC) or 1% DMSO and irradiated with 405‐nm light (87.6 J cm^−2^). The results are the average of at least three independent replicates ± the standard deviation. F) DPH fluorescence of *C. albicans* cells treated with 1% DMSO or different MMs (0.5–2× MIC) and irradiated with 405‐nm light (87.6 J cm^−2^). Amphotericin B (AMB) was used as a control. G) Effect of exogenous ergosterol, plasma membrane phospholipids (phosphatidylethanolamine, PE, and phosphatidylcholine, PC), or mitochondrial phospholipids (phosphatidylglycerol, PG, and cardiolipin, CL) on the sensitivity of *C. albicans* to MMs, evaluated as the MIC, in the presence of 405‐nm light (87.6 J cm^−2^). Symbols denote the average of three replicas. Asterisks denote the significance of the differences in pairwise comparisons between the MIC in the absence and the presence of increasing concentrations of different exogenous phospholipids. H) SEM images of *C. albicans* treated with 1% DMSO or 0.5× MIC of visible‐light‐activated MM **1**. I) TEM images of *C. albicans* treated with 1% DMSO or 0.5× MIC of visible‐light‐activated MM **1**. Arrowheads indicate enlarged mitochondria in MM‐treated samples compared with normal mitochondria in DMSO‐treated samples (arrows). The bar indicates the scale. Unless otherwise indicated, the results for MMs and DMSO are always reported in the presence of light. J) Confocal microscopy images of *C. albicans* treated with MM **1** (8 µm) and then labeled with the fluorescent mitochondrial dye MitoTracker Green (10 nm) and the fluorescent plasma membrane dye FM 4–64 (40 nm). The image identified as “combined” is a merger of the natural fluorescence of MM **1**, MitoTracker Green, and FM 4–64. The bar indicates the scale. K) Box‐and‐whisker plot of the percentage overlap of fluorescence from MitoTracker Green or FM 4–64 with the natural fluorescence from MM **1**. Light was omitted in colocalization experiments. Results are shown as the average of five independent cells ± the standard deviation. Asterisks denote the significance of the differences in pairwise comparisons with 1% DMSO controls performed in GraphPad prism. *p <0.05, **p <0.01, ***p <0.001, ****p <0.0001.

The fluorescence of the nucleic acid‐binding dye propidium iodide (PI) was used to determine the effects of MMs on plasma membrane integrity. Treatment with visible‐light‐activated MMs resulted in increased PI uptake (**Figure** **2**A), particularly at 0.5× MIC (*p* <0.05) (Figure 2B), indicating MM‐induced plasma membrane permeabilization. Impaired plasma membrane integrity was also evidenced by decreased intracellular calcein fluorescence (Figure 2C) in cells treated with increasing MM concentrations (Figure 2D). Additionally, MM treatment significantly increased the extracellular adenosine triphosphate (ATP) concentration (*p* <0.05) (Figure 2E), reflecting intracellular content leakage.

This initial observation prompted us to investigate whether MMs act directly on the fungal plasma membrane by monitoring the fluorescence of 1,6‐diphenyl‐hexa‐1,3,5‐triene (DPH),^[^ ^20^ ^]^ which has a high affinity for membrane phospholipids. In contrast to AMB, which binds plasma membrane ergosterol and reduces DPH fluorescence (Figure 2F, *p* <0.05), treatment with MM had no effect on DPH fluorescence (Figure 2F), indicating that MMs do not bind plasma membrane phospholipids of *C. albicans*.

The binding of MM to the fungal plasma membrane was further investigated in competition binding assays with exogenous ergosterol, the main fungal sterol, or phosphatidylethanolamine and phosphatidylcholine, the main phospholipids of the fungal plasma membrane. Treatment with increasing concentrations of ergosterol resulted in a reduction in MM MIC, whereas phosphatidylethanolamine and phosphatidylcholine either had no significant effect or caused only a small increase in MM MIC (Figure 2G), confirming that MMs do not bind the plasma membrane sterols or phospholipids of *C. albicans*. Similarly, exogenous glucose‐6‐phosphate, representing negatively charged fungal cell wall polysaccharides, did not affect MM MIC (Figure S3, Supporting Information), and sorbitol did not offer protection against MM‐induced growth arrest (Figure S4, Supporting Information), indicating that the fungal cell wall is also not targeted by MMs.

Scanning electron microscopy confirmed that MM treatment did not alter the cell surface of *C. albicans* (Figure 2H). Conversely, transmission electron microscopy (TEM) revealed extensive intracellular structural damage in MM‐treated *C. albicans*, characterized by the loss of most subcellular membrane systems (Figure 2I). Competition binding experiments with the negatively charged mitochondrial phospholipids cardiolipin and phosphatidylglycerol revealed a substantial increase in MM MIC (up to 512‐fold) (Figure 2G), suggesting that MMs bind these phospholipids.

**Figure 3:**
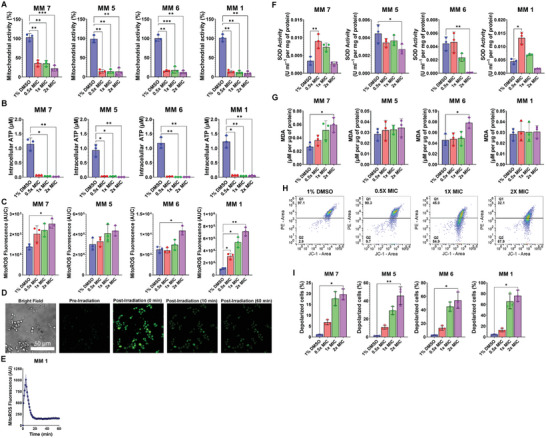
Visible‐light‐activated MMs trigger mitochondrial dysfunction and oxidative stress. A) Mitochondrial dehydrogenase activity in *Candida albicans* treated with 1% DMSO or different MMs (0.5–2 × MIC) in the presence of 405‐nm light (87.6 J cm^−2^). B) Intracellular ATP levels in *C. albicans* treated with 1% DMSO or different MMs (0.5–2× MIC) and 405‐nm light (87.6 J cm^−2^). C) Mitochondrial ROS levels detected by spectrofluorimetry using the MitoROS 580 probe in *C. albicans* treated with 1% DMSO or different MMs (0.5–2× MIC) and 405‐nm light (87.6 J cm^−2^). D) Mitochondrial ROS levels detected by confocal microscopy using the MitoROS 580 probe in *C. albicans* treated with MM **1** (1× MIC) before and after light activation under the microscope. The bar indicates the scale. E) Temporal profile of MitoROS 580 fluorescence detected by confocal microscopy, shown as the average fluorescence intensity (line) and standard error of the mean (shaded area). F) SOD activity normalized to the protein content in *C. albicans* treated with 1% DMSO or different MMs (0.5–2× MIC) and 405‐nm light (87.6 J cm^−2^). G) Lipid peroxidation assessed from malondialdehyde levels (MDA) normalized by protein content in *C. albicans* treated with 1% DMSO or different MMs (0.5–2× MIC) and 405‐nm light (87.6 J cm^−2^). H) Representative shifts in the fluorescence of JC‐1 in *C. albicans* treated with 1% DMSO or MM **1** (0.5–2× MIC) and 405‐nm light (87.6 J cm^−2^) detected by flow cytometry denoting MM‐induced depolarization of the mitochondrial membrane. I) Changes in the percentage of depolarized cells in *C. albicans* treated with 1% DMSO or different MMs (0.5–2× MIC) and 405‐nm light (87.6 J cm^−2^) detected with JC‐1 by flow cytometry. All results are shown as the average of at least three independent replicates ± the standard deviation. Asterisks denote the significance of the differences in pairwise comparisons with 1% DMSO controls performed in GraphPad Prism. *p <0.05, **p <0.01, ***p <0.001, ****p <0.0001. Unless otherwise stated, the results for MMs and DMSO are always reported in the presence of light.

This observation prompted us to investigate whether MMs target mitochondria. The cellular distribution of MM **1** (the most potent MM) in *C. albicans* was examined by confocal microscopy, which revealed that MM **1** was internalized within cells (Figure 2J). Image analysis confirmed an average areal colocalization of MM **1** and the mitochondrial dye MitoTracker Green fluorescence of 52.5%, whereas that of MM **1** with the plasma membrane dye N‐(3‐Triethylammoniumpropyl)‐4‐(6‐(4‐(Diethylamino) Phenyl) Hexatrienyl) Pyridinium Dibromide (FM 4–64) was 5.2% (Figure 2K, *p* <0.01). Investigating the effects of visible‐light‐activated MMs on mitochondrial function revealed a 67–92% reduction (*p* < 0.01) in mitochondrial dehydrogenase activity in MM‐treated cells compared with DMSO controls (**Figure** **3**A). Intracellular ATP levels were also significantly decreased (*p* <0.05) following MM treatment, from ≈1 µm in untreated samples to ≈0.005 µm in 2 × MIC‐treated samples (Figure 3B). Based on these results, we shifted our focus to investigating the effects of MM‐induced mechanical disruption on intracellular processes.

A significant (*p* <0.05) and concentration‐dependent increase in mitochondrial reactive oxygen species (ROS) levels (up to 7‐fold) was observed in MM‐treated samples using the mitochondrial superoxide‐sensitive probe MitoROS 580 (Figure 3C). Confocal microscopy revealed a sharp increase in ROS levels in irradiated MM **1**‐treated samples (Figure 3D), which rapidly returned to preexposure levels after irradiation cessation (Figure 3E), possibly reflecting mitochondrial tolerance to sublethal superoxide levels. Accordingly, cells treated with 0.5 × MIC MM **7** and MM **1** displayed increased superoxide dismutase (SOD) activity (Figure 3F, *p* <0.05). However, the mitochondrial antioxidant capacity was eventually exhausted, resulting in oxidative damage to biomolecules, as evidenced by increased levels of the lipid peroxidation product malonaldehyde in cells treated with 2 × MIC MM **6** and MM **7** (Figure 3G). MM treatment also decreased mitochondrial membrane potential (Figure 3H), as measured by the shift in 5,5′,6,6′‐tetrachloro‐1,1′,3,3′‐tetraethylbenzimidazolocarbocyanine iodide (JC‐1) fluorescence, in a concentration‐dependent manner, with up to 75% of cells depolarized after MM treatment (Figure 3I, *p* <0.05).

Together, these results identify bioenergetic deficit and oxidative stress, resulting in mitochondrial membrane depolarization, as important contributors to the antifungal mechanism of action of visible‐light‐activated MMs. However, cells depleted of ATP by chemically induced de‐energization (Figure S5, Supporting Information) or electron transport chain inhibition (Figure S6, Supporting Information) were as susceptible to MM‐induced killing as energized cells, demonstrating that energy depletion alone cannot explain the MM killing mechanism. Likewise, cells pre‐depolarized with carbonyl cyanide 3‐chlorophenylhydrazone or carbonyl cyanide 4‐(trifluoromethoxy)phenylhydrazone could still be killed by visible‐light‐activated MMs (Figure S7, Supporting Information). Moreover, fermentative growth did not protect against MM‐mediated death (Figure S8, Supporting Information), unlike antifungals that induce mitochondrial dysfunction by collapsing the mitochondrial membrane potential.^[^ ^21^, ^22^ ^]^ These results indicate that mitochondrial membrane depolarization alone also cannot explain MM‐induced death. Additionally, the mitigation of MM‐induced killing by the iron scavenger 2,2′‐dipyridyl (Figure S9A, Supporting Information) could be ascribed to its effect on the growth rate (Figure S9B, Supporting Information) because it did not impact MM‐induced ROS generation (Figure S9C, Supporting Information). Conversely, the mitochondrial superoxide scavenger MitoTEMPO reduced ROS generation (Figure S10A, Supporting Information) but did not affect survival following MM treatment (Figure S10B, Supporting Information).

**Figure 4:**
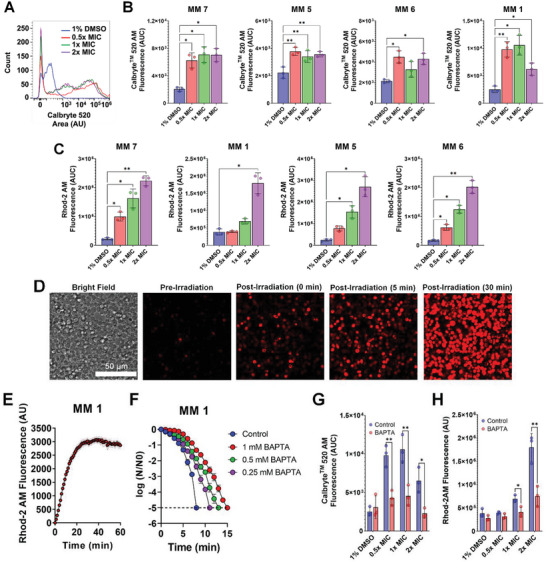
Visible‐light‐activated MMs elicit intracellular calcium overload. A) Representative histograms of Callbryte 520 AM fluorescence used to detect cytosolic calcium levels in *Candida albicans* treated with increasing concentrations of MM **1** or 1% DMSO in the presence of 405‐nm light (87.6 J cm^−2^) by flow cytometry. B) Cytosolic calcium levels detected with Callbryte 520 AM by spectrofluorimetry in *C. albicans* treated with increasing concentrations of different MMs (0.5–2× MIC) or 1% DMSO in the presence of 405‐nm light (87.6 J cm^−2^). C) Mitochondrial calcium levels detected with Rhod‐2 AM by spectrofluorimetry in *C. albicans* treated with increasing concentrations of different MMs (0.5–2× MIC) or 1% DMSO in the presence of 405‐nm light (87.6 J cm^−2^). D) Mitochondrial calcium levels detected with Rhod‐2 AM by confocal microscopy in *C. albicans* treated with MM **1** (1× MIC) before and after light activation. E) Temporal profile of Rhod‐2 AM fluorescence detected by confocal microscopy, shown as the average fluorescence intensity (line) and standard error of the mean (shaded area). F) Effect of different concentrations (0.25–1.25 mm) of the intracellular calcium chelator BAPTA‐AM on the killing of *C. albicans* by MM **1** (2× MIC). Killing was assessed as the reduction in colony‐forming units (CFU), expressed as the logarithm of base 10 of the ratio between the CFU at each time point (*N*) and the CFU at time zero (*N*0). The results are expressed as the average of at least three replicates ± the standard error of the mean. The dashed line denotes the limit of detection of the method. G) Cytosolic calcium levels detected by spectrofluorimetry with Callbryte 520 AM in *C. albicans* amended with 1.25 mm BAPTA‐AM and then treated with increasing concentrations of MM **1** or 1% DMSO in the presence of 405‐nm light (87.6 J cm^−2^). H) Cytosolic calcium levels detected with Rhod‐2 AM by spectrofluorimetry in *C. albicans* amended with 1.25 mm BAPTA‐AM and then treated with increasing concentrations of MM **1** or 1% DMSO in the presence of 405‐nm light (87.6 J cm^−2^). The results are the average of at least three independent replicates ± the standard deviation. Asterisks denote the significance of the differences in pairwise comparisons with 1% DMSO controls performed in GraphPad Prism. *p <0.05, **p <0.01, ***p <0.001, ****p <0.0001. Unless otherwise stated, the results for MMs and DMSO are always reported in the presence of light.

In addition to their roles in energy production and ROS generation, mitochondria are crucial for calcium homeostasis and apoptosis.^[^ ^23^ ^]^ Therefore, we investigated whether these processes could also contribute to the MM mechanism of action. MM‐treated cells showed increased cytosolic calcium levels detected with the Calbryte 520 AM fluorescent probe (**Figure** **4**A) of up to fourfold (*p* <0.05) (Figure 4B). Mitochondrial calcium levels detected using the fluorescent probe Rhod‐2 AM showed an even greater increase (up to twelvefold, *p* <0.05) in MM‐treated cells (Figure 4C), which was also evident by live‐cell calcium imaging using confocal microscopy (Figure 4D,E). Mitigation of MM‐induced cell death (Figure 4F) and the MM‐induced increases in cytosolic (Figure 4G) and mitochondrial calcium (Figure 4H) by the calcium chelator 1,2‐bis(2‐aminophenoxy)ethane‐*N*,*N*,*N*′,*N*′‐tetraacetic acid acetoxymethyl ester (BAPTA‐AM) confirmed the importance of calcium homeostasis in the antifungal mechanism of action of MMs.

**Figure 5:**
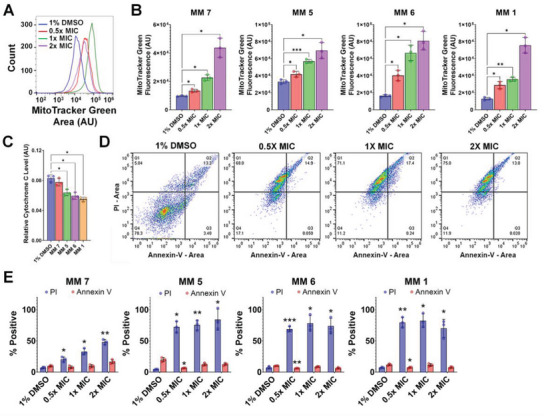
Visible‐light‐activated MMs cause mitochondrial swelling, release of mitochondrial cytochrome c, and necrosis. A) Representative histograms of MitoTracker Green fluorescence in *Candida albicans* treated with 1% DMSO or MM **1** (0.5–2× MIC) and 405‐nm light (87.6 J cm^−2^) detected by flow cytometry. B) Altered mitochondrial mass/volume determined from changes in MitoTracker Green fluorescence detected by flow cytometry in *C. albicans* treated with 1% DMSO or different MMs (0.5–2× MIC) and 405‐nm light (87.6 J cm^−2^). C) Mitochondrial cytochrome c levels in *C. albicans* treated with 1% DMSO or different MMs (2× MIC) and 405‐nm light (87.6 J cm^−2^). D) Representative changes in the percentage of PI‐positive/negative and Annexin V‐positive/negative cells in *C. albicans* treated with 1% DMSO or MM **1** (0.5–2× MIC) and 405‐nm light (87.6 J cm^−2^) detected by flow cytometry. E) Percentage of PI‐positive and Annexin V‐positive cells in *C. albicans* treated with different MMs (0.5–2 × MIC) or 1% DMSO and 405‐nm light (87.6 J cm^−2^) detected by flow cytometry. The results are the average of at least three independent replicates ± the standard deviation. Unless otherwise indicated, the results for MMs and DMSO are always reported in the presence of light. Asterisks denote the significance of the differences in pairwise comparisons with 1% DMSO controls performed in GraphPad prism. *p <0.05, **p <0.01, ***p <0.001, ****p <0.0001.

MM‐treated cells showed increased MitoTracker Green fluorescence (**Figure** **5**A), particularly at 2 × MIC (Figure 5B, *p* <0.05), denoting increased mitochondrial mass/volume. This finding may be due to water influx into mitochondria following calcium overload, consistent with the substantial increase in mitochondrial size in MM‐treated cells compared with DMSO controls detected by TEM (Figure 2I). Additionally, significant reductions in mitochondrial cytochrome c levels (*p* <0.05) were observed in cells treated with 2 × MIC of MMs **1**, **5**, and **6** (Figure 5C), suggesting mitochondrial outer membrane rupture and intramitochondrial content leakage.

An Annexin V‐based assay was used to investigate whether the previously described MM‐induced physiological changes lead to cell death by apoptosis or necrosis.^[^ ^24^ ^]^ *C. albicans* protoplasts treated with MM (0.5–2 × MIC) or 1% DMSO and irradiated with 405‐nm light (87.6 J cm^−2^) were labeled with Annexin V and PI and analyzed by flow cytometry (Figure 5D). The results confirmed that MM treatment induced cell death by necrosis, as evidenced by a significant increase in the percentage of PI‐positive protoplasts by up to 80% (*p* <0.01), but only a non‐significant change in the percentage of Annexin V‐positive protoplasts (Figure 5E).

### 2.3 MMs Potentiate the Activity of Conventional Antifungals

**Figure 6:**
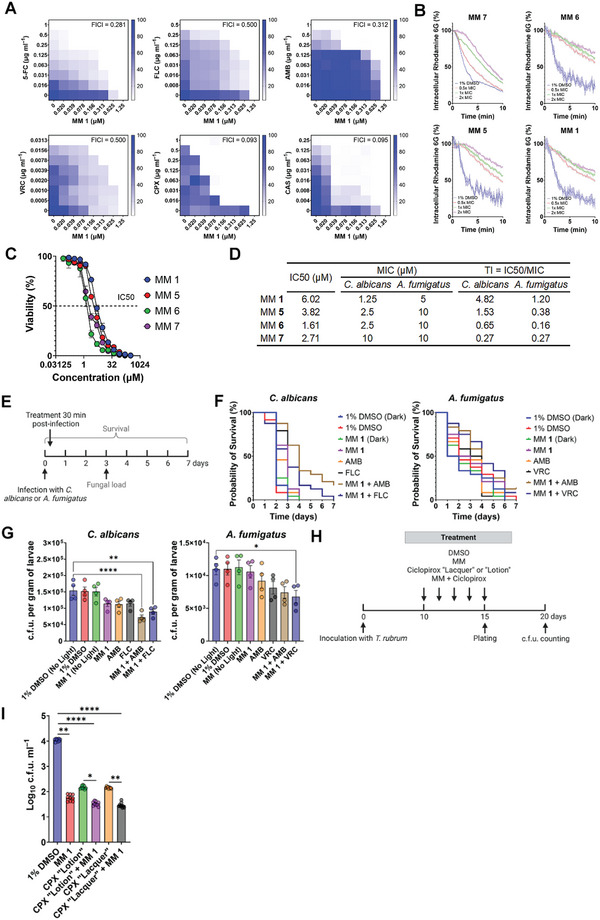
Visible‐light‐activated MMs synergize with conventional antifungals in vitro, in vivo, and ex vivo. A) Representative checkerboard patterns showing the interaction between visible‐light‐activated MM **1** and various conventional antifungal drugs in *Candida albicans* and the respective fractional inhibitory concentration indices (FICI) for the interaction between MM **1** and each antifungal. The results are shown as a heatmap, with the white color denoting no growth (0%) and the blue color denoting growth (100%). Results are the average of three independent replicates. Growth was assessed as the absorbance at 630 nm. 5‐FC: 5‐Fluorocytosine. AMB: Amphotericin B. FLC: Fluconazole. VRC: Voriconazole. CAS: Caspofungin. CPX: Ciclopirox. B) Decrease in intracellular rhodamine 6G fluorescence, used to assess energy‐dependent efflux pump activity, in *C. albicans* treated with increasing concentrations of different MMs (0.5–2× MIC) or 1% DMSO in the presence of 405‐nm light (87.6 J cm^−2^). The lines represent the average of at least three independent replicates, and the shaded area represents the standard error. Unless otherwise noted, the results for MMs and DMSO are always reported in the presence of light. C) Effect of increasing concentrations of different MMs plus 405‐nm light (87.6 J cm^−2^) on the viability of a mammalian cell line (HEK293T). The dashed line indicates the IC_50_, that is, the concentration of MM that results in a 50% reduction in cell viability. Results are the average of three independent replicates. D) Therapeutic index (TI) calculated as the ratio between the MIC for each MM in *C. albicans* and *Aspergillus fumigatus* and their respective IC_50_ values. E) Workflow used to study the anti‐infective activity of MMs in vivo. Created in Biorender.com. F) Survival curves of worms infected with *C. albicans* or *A. fumigatus* subjected to monotherapy with visible‐light‐activated MM **1** (1× MIC plus 405‐nm light at 87.6 J cm^−2^), conventional antifungal agents (1× MIC) or combination therapy with visible‐light‐activated MM **1** (1× MIC plus 405‐nm light at 87.6 J cm^−2^) followed by treatment with conventional antifungals (1× MIC). Data represent pooled results from three independent biological replicates, each containing eight individuals (*n* = 24). G) Fungal load of worms (*n* = 4) infected with *C. albicans* or *A. fumigatus* subjected to monotherapy with visible‐light‐activated MM **1** (1× MIC plus 405‐nm light at 87.6 J cm^−2^), conventional antifungal agents (1× MIC), or combination therapy with visible‐light‐activated MM **1** (1× MIC plus 405‐nm light at 87.6 J cm^−2^) followed by treatment with conventional antifungal agents (1× MIC) 48 h after infection. H) Workflow used to study the anti‐infective activity of MMs ex vivo. Created in Biorender.com. I) Fungal load of porcine nail samples (*n* = 9) infected with *Trichophyton rubrum* and subjected to five consecutive rounds of monotherapy with visible‐light‐activated MM **1** plus 405‐nm light at 87.6 J cm^−2^, different topical formulations of the conventional antifungal ciclopirox (“Lotion” and “Lacquer”) or combination therapy with visible‐light‐activated MM **1** plus 405‐nm light at 87.6 J cm^−2^ followed by treatment with a conventional antifungal agent. Asterisks denote the significance of the differences in pairwise comparisons performed in GraphPad prism. *p <0.05, **p <0.01, ***p <0.001, ****p <0.0001. Unless otherwise stated, the results for MMs and DMSO are always reported in the presence of light.

A modified checkerboard assay was used to study the interaction of visible‐light‐activated MMs with conventional antifungals in *C. albicans*. Cells were treated with increasing concentrations of MMs (up to 1× MIC), irradiated with 405‐nm light (87.6 J cm^−2^), and then challenged with increasing concentrations of different antifungals (up to 1× MIC, Table S2, Supporting Information). The type of interaction between MMs and conventional antifungals was assessed by calculating the fractional inhibitory concentration index (FICI), with a FICI of ≤ 0.5, 0.5 < *x* ≤ 4, or > 4, denoting synergistic, additive, or antagonistic interactions, respectively.^[^ ^25^ ^]^ MM **1** synergized with all antifungals tested (**Figure** **6**A), with FICIs ranging from 0.093 (MM **1**–ciclopirox) to 0.500 (MM **1**–FLC and MM **1–**voriconazole [VRC]).

Rhodamine 6G efflux was used to assess whether the potentiation of conventional antifungals by MMs was due to the impaired activity of energy‐dependent efflux pumps. DMSO controls effluxed 75–85% of the accumulated rhodamine 6G, whereas MM‐treated cells effluxed only 31–68% (Figure 6B), denoting the interference of MMs with the activity of efflux pumps.

### 2.4 MMs Potentiate Conventional Antifungals In Vivo and Ex Vivo

The toxicity of visible‐light‐activated MMs to mammalian cells was investigated in human embryonic kidney cells (HEK293T) treated with increasing MM concentrations and 87.6 J cm^−2^ of 405‐nm light. Vehicle‐treated controls exposed to this light dose showed only a non‐significant reduction in cell viability (Figure S11, Supporting Information). The MM concentration that reduced viability by 50%, or half maximal inhibitory concentration (IC_50_), calculated from dose‐response curves (Figure 6C), ranged from 1.61–6.02 µm (Figure 6D). The IC_50_ and MIC were used to calculate the therapeutic index. With a therapeutic index ≥1 (Figure 6D), MM **1** was used for in vivo and ex vivo studies.

The in vivo antibacterial activity of MM **1** was evaluated in a *Galleria mellonella* model of systemic infection with *C. albicans* or *A. fumigatus*. Infected worms were treated with 1% DMSO or MM **1** (1× MIC) with or without light or with conventional antifungals (1× MIC), namely, the polyene AMB and the azole FLC (*C. albicans*) or VRC (*A. fumigatus*). The effect of dual therapy combining light‐activated MM **1** (1× MIC) and conventional antifungals (AMB or azole, 1× MIC) was also evaluated. Worm survival was monitored for 7 days, and fungal burden was assessed in a larval subset 48 h post‐infection (Figure 6E).

All *C. albicans*‐infected worms treated with DMSO, MM, single antifungals, or MM plus FLC died within 3 days (Figure 6F). However, MM **1** + AMB significantly improved survival compared with individual treatments (*p* <0.0001), with ≈17% of worms surviving to day 7 (Figure 6F and Table S4, Supporting Information). A significant reduction (*p* <0.01) in fungal burden was also observed in worms subjected to combination therapy compared with DMSO controls (Figure 6G).

In *A. fumigatus‐*infected worms, dual therapy (MM **1** plus antifungal) also improved survival compared with untreated samples (Figure 6F). Moreover, MM **1** + VRC significantly reduced (*p* <0.05) worm fungal burden compared with DMSO controls (Figure 6G). However, statistically significant differences in the survival of worms subjected to dual therapy versus MM or antifungal alone were not detected (Table S5, Supporting Information).

The ability of MMs to reduce fungal burden in mammals was investigated using an ex vivo onychomycosis porcine model infected with a strain of *T. rubrum* (ATCC 10 218) isolated from a human onychomycosis case. *T. rubrum*‐infected porcine nails were treated with 1% DMSO or MM **1** alone (0.77% (w/v) in DMSO) plus 405‐nm light (87.6 J cm^−2^) or two formulations of the topical synthetic hydroxypyridone ciclopirox: a 0.77% “lotion” and an 8% “lacquer.” The effect of dual therapy (MM **1** plus ciclopirox) was also evaluated. The fungal load was assessed 5 days post‐treatment (Figure 6H). Compared with DMSO controls, MM **1** alone significantly reduced fungal burden by ≈2 log_10_ (Figure 6I). Dual therapy (MM **1** + ciclopirox) performed significantly better than ciclopirox alone (*p* <0.001) but did not outperform MM **1** alone (Figure 6I and Table S6, Supporting Information).

## 3 Discussion

Here, we report the ability of synthetic 405‐nm‐visible‐light‐activated MMs to kill unicellular and multicellular planktonic fungi (Figure 1C,D). At up to 2× MIC, killing depended entirely on light activation of the fast rotation rates of MMs (Figure 1D and Figure S1, Supporting Information) and could be remotely controlled by adjusting the light dose, with higher light doses enhancing antifungal activity (Figure 1F). In contrast to conventional antifungals, MM MIC remained stable over 20 cycles of repeated treatment (Figure 1I), suggesting that resistance to MMs is not easily achieved.

In addition to planktonic cells, light‐activated MMs were also able to rapidly eliminate established biofilms of *C. albicans*, reducing both biofilm viability (Figure 1G) and biomass (Figure 1H) within minutes of light activation more efficiently than AMB for the same treatment time. Similar results were observed following the treatment of biofilms of *S. cerevisiae* with light‐activated MMs (Figure S12, Supporting Information). Members of the *Candida* genus are the most common fungal species associated with biofilm infections of medical devices,^[^ ^26^ ^]^ and biofilm formation is an important process associated with *C. albicans* virulence.^[^ ^27^ ^]^ Bacteria in a biofilm can also detach from biological or artificial surfaces, enter the bloodstream, and migrate to other parts of the body through the process of hematogenous dissemination, leading to candidemia and septicemia. Fungal biofilms are highly resistant to antifungal drugs and host immune defenses, making the treatment of biofilm‐associated infections particularly challenging.^[^ ^26^ ^]^ The observed reduction in biofilm biomass and viability after treatment with MMs suggests that the molecules are not only capable of physically destroying the extracellular polymeric matrix of the biofilm but also killing fungal cells within the biofilm. Future studies will be required to investigate whether MMs can also attenuate other processes that contribute to *C. albicans* pathogenicity including filamentation, yeast‐to‐hyphae transition, and surface adhesion.^[^ ^27^ ^]^

Mechanism of action studies in *C. albicans* showed that MMs bind the negatively charged mitochondrial phospholipids cardiolipin and phosphatidylglycerol (Figure 2G), and confocal microscopy confirmed substantial (52.5%) colocalization of MMs with mitochondria (Figure 2J,K), identifying mitochondria as the main cellular targets of MMs in fungi. Since the light was omitted during colocalization experiments, binding of MMs to mitochondrial phospholipids occurs in the dark, possibly through supramolecular interactions between the positively charged MM amine groups after protonation at biological pH (Figure 1C) and the negatively charged phosphate groups of cardiolipin and phosphatidylglycerol. However, binding of MMs to mitochondria alone is not overtly detrimental (Figure S13, Supporting Information), and light must activate the rapid rotation of MMs bound to mitochondrial phospholipids to trigger antifungal activity.

The identification of cardiolipin and phosphatidylglycerol as MM targets reconciles our findings and previous observations on the broad spectrum of biological activity of MMs, ranging from bacteria^[^ ^15^ ^]^ to mammalian cells,^[^ ^14^ ^]^ as these phospholipids are common crucial components of all these organisms. Phosphatidylglycerol and cardiolipin are major components of the bacterial membrane but are mainly found in the mitochondrial membranes of eukaryotes, consistent with their endosymbiotic origin.^[^ ^28^ ^]^ The distinct locations of these phospholipids in different organisms explain why MMs cause substantial damage to bacterial membranes^[^ ^15^ ^]^ but produce predominantly intracellular effects in *C. albicans*.

By stabilizing the electron transport chain, cardiolipin is critical for mitochondrial function, and yeasts deficient in cardiolipin show impaired mitochondrial bioenergetics.^[^ ^29^ ^]^ Therefore, binding of MMs to mitochondrial phospholipids and their subsequent activation by light could affect normal mitochondrial processes, as shown by decreased mitochondrial activity (Figure 3A), intracellular ATP (Figure 3B), and mitochondrial membrane potential (Figure 3H,I), as well as increased mitochondrial superoxide radical formation (Figure 3C–E) in MM‐treated cells.

In addition to their role in energy and ROS generation, in higher eukaryotes, mitochondria also modulate cellular calcium homeostasis due to their proximity to the endoplasmic reticulum, the main calcium reservoir.^[^ ^30^ ^]^ In yeast, the vacuole is the primary cellular calcium storage organelle, and the role of mitochondria in calcium homeostasis is unclear because there is no mitochondrial calcium uniporter or calcium‐sensitive dehydrogenases.^[^ ^31^ ^]^ However, calcium enters yeast mitochondria when cytosolic calcium levels increase,^[^ ^32^ ^]^ and free fatty acids from mitochondrial phospholipid degradation have been shown to activate vigorous mitochondrial Ca^2+^:2H^+^ antiporter activity.^[^ ^33^ ^]^ The observations that MM treatment significantly increased intracellular calcium levels (Figure 4B–E) and that calcium chelation attenuated MM‐induced killing (Figure 4F) by lessening the MM‐induced intracellular calcium increase (Figure 4G,H) provide compelling evidence that calcium overload is involved in the antifungal mechanism of action of MMs.

Elevated intracellular calcium levels in MM‐treated cells can be attributed to intracellular ATP depletion (Figure 3B) resulting from mitochondrial dysfunction. Since intracellular calcium homeostasis depends on ATPases in the plasma membrane, vacuole, and other organelles,^[^ ^34^ ^]^ ATP depletion leads to uncontrolled calcium uptake from the extracellular medium and its release from intracellular stores. This is followed by water influx leading to swelling of the cell and organelles, including mitochondria (Figure 5B), which eventually burst and release the intramitochondrial contents into the cytoplasm, as indicated by a significant decrease in mitochondrial cytochrome C concentration in MM‐treated cells.

**Figure 7:**
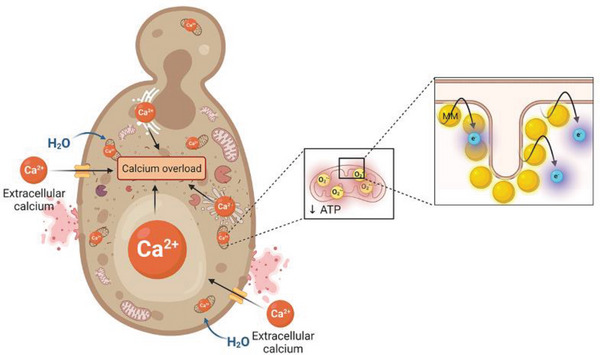
Schematic representation of the mechanisms of action of antifungal MMs. MMs bind cardiolipin and phosphatidylglycerol in the inner mitochondrial membrane, destabilizing the electron transport chain. This leads to increased electron leakage and superoxide radical formation, causing oxidative stress. Consequently, ATP synthesis and mitochondrial membrane potential are reduced. ATP‐dependent calcium transporters in the plasma membrane and intracellular organelles stop functioning, leading to increased cytosolic calcium levels, which activate calcium‐dependent degradative enzymes. Increased water influx ensues, leading to swelling of organelles, which eventually burst, releasing even more degradative enzymes and intramitochondrial contents to the cytoplasm. Eventually, the integrity of the plasma membrane is compromised, and intracellular contents leak out of the cell. Created in Biorender.com.

Damage to the plasma membrane, intracellular ATP depletion, leakage of cell contents, and swelling of mitochondria are common features of necrotic death.^[^ ^35^ ^]^ The necrotic nature of MM killing was confirmed by the significant increase in the percentage of necrotic but not apoptotic cells after MM treatment (Figure 5D,E). Overall, MM‐induced fungal cell death via necrosis results from the cumulative effects of oxidative stress and bioenergetic deficit triggered by light activation of MMs bound to mitochondrial phospholipids, leading to calcium overload and osmotic shock (**Figure** **7**). Because these processes occurred in *C. albicans* and *S. cerevisiae* (Figure S14, Supporting Information), the proposed antifungal mechanism of action of MMs appears to be conserved in yeast.

Unlike most conventional antifungals, which act on a single target in the cell, the involvement of widespread mitochondrial dysfunction and calcium overload in the mechanism of action of antifungal MMs may explain the inability to detect the development of resistance to MM treatment, as this damage cannot in principle be mitigated by one or a few concurrent mutations. Since MMs bind cardiolipin and phosphatidylglycerol and yeasts lacking both phospholipids are severely impaired or not viable,^[^ ^36^ ^]^ simultaneous mutations in both phospholipids that could prevent MM binding and lead to resistance are unlikely. Further studies are needed to understand the precise interactions between MMs and cardiolipin and phosphatidylglycerol in order to assess the extent to which mutations leading to subtle changes in the conformation and/or composition of these phospholipids might affect sensitivity to MM‐induced killing.

Importantly, the calcium dysfunction triggered by MMs is distinct from that involved in azole resistance.^[^ ^37^ ^]^ This is evidenced by the opposite role of calcium chelation and calcineurin in the action of azoles^[^ ^37^, ^38^, ^39^ ^]^ compared with that of MMs (Figure 4F and Figure S15, Supporting Information), which explains the lack of cross‐resistance between MMs and azoles (Table S3, Supporting Information).

In addition to their direct antifungal activity, visible‐light‐activated MMs synergized with conventional antifungals in *C. albicans* (Figure 6A) and in *S. cerevisiae* (Figure S16, Supporting Information). This may be due to the orthogonal targeting of different cellular processes by MMs and conventional antifungals.^[^ ^40^ ^]^ Photoinactivation of catalase by blue light^[^ ^41^ ^]^ may also sensitize cells to the deleterious effects of MMs. Moreover, the fluorescence of rhodamine 6G, a substrate of some of the energy‐dependent efflux pumps whose overexpression has been associated with azole resistance,^[^ ^42^, ^43^ ^]^ showed a significant decrease in MM‐treated cells (Figure 6B). These results suggest that MMs also enhance the effect of conventional antifungal drugs by impairing the activity of energy‐dependent efflux pumps. Enhanced efflux is an important mechanism by which microorganisms attenuate the effect of antimicrobials by reducing the amount of drug that accumulates in the cell.^[^ ^44^ ^]^ Accordingly, inhibition of efflux pumps has been found to enhance the activity of antifungal drugs by increasing their intracellular levels.^[^ ^45^ ^]^ The observed impairment of the activity of energy‐dependent efflux pumps by MMs can be attributed to the MM‐induced decrease in intracellular ATP content (Figure 3B), which is consistent with the previously reported increase in the azole susceptibility of cells deprived of energy.^[^ ^46^ ^]^

In vivo studies on the antifungal efficacy of MMs were performed on *G. mellonella*. *G. mellonella* is a simple invertebrate that has been used extensively as a model system for studying the in vivo efficacy of antifungal agents against *C. albicans* ^[^ ^47^ ^]^ and *A. fumigatus*.^[^ ^48^ ^]^ *G. mellonella* does not have adaptive immunity, but its innate immune system has similarities to that of vertebrates in terms of function and anatomy.^[^ ^49^ ^]^ Importantly, pathogenicity in mice and *G. mellonella* models of infections is correlated,^[^ ^48^, ^50^ ^]^ suggesting that findings from studies with *G. mellonella* are translatable to vertebrates.

Dual therapy of *C. albicans*‐ or *A. fumigatus*‐infected worms with light‐activated MMs and conventional antifungals improved survival (Figure 6F) and reduced fungal burden (Figure 6G) compared with vehicle‐treated controls. In *C. albicans*, combination therapy with AMB and MM significantly improved survival compared with treatment with AMB or MM alone, suggesting a synergistic interaction between these antimicrobial modalities in vivo. Similarly, MM **1** potentiated the activity of the commonly prescribed antifungal agent ciclopirox^[^ ^51^ ^]^ in an ex vivo onychomycosis porcine model (Figure 6I).

Most conventional antifungal agents, such as AMB, exhibit severe toxicity leading to undesirable side effects.^[^ ^4^ ^]^ A therapeutic approach combining sublethal MMs to sensitize cells to conventional antifungals could mitigate the side effects of existing antifungal therapies. Moreover, the observation that MMs not only kill fungal cells directly but can also enhance the effect of conventional antifungal drugs by targeting a distinct process in the cell (i.e., intracellular calcium homeostasis) and/or preventing their efflux identifies MMs as dual mode‐of‐action antifungals that could provide a much‐needed new therapeutic option to combat pan‐resistant fungal strains such as *C. auris*,^[^ ^8^ ^]^ for which there are currently limited treatment options. MMs with improved safety profiles that specifically target fungal mitochondria can be developed by exploiting differences in the chemical composition of fungal and mammalian mitochondrial phospholipids^[^ ^52^ ^]^ and/or by modifying MMs with peptide addends that target mitochondrial proteins found in fungi but not in mammals such as the fungal‐type II nicotinamide adenine dinucleotide (NADH) dehydrogenases.^[^ ^53^ ^]^

## 4 Experimental Section

### Synthetic Chemistry

Details on the synthesis and characterization of MM **7** are provided in Supporting Information. Information on the synthesis and characterization of the other MMs investigated in this study can be found elsewhere.^[^ ^15^ ^]^

### Strains and Reagents

Five fungal strains were used in this study: the yeast *S. cerevisiae* (ATCC 13007^TM^), the yeast‐like fungus *C. albicans* (ATCC 18804^TM^), and the molds *A. fumigatus* (ATCC 1022^TM^), *M. gypseum* (ATCC 10215^TM^), and *T. rubrum* (ATCC 10218^TM^). All fungi were obtained from ATCC (Manassas, VA, USA).

Unless otherwise noted, all chemicals were purchased from MedChem Express (Princeton, NJ, USA), Caymanchem (Ann Arbor, MI, USA), or Millipore‐Sigma (St. Louis, MO, USA) and prepared in 100% DMSO or an appropriate solvent, per the distributor's instructions.

### Antifungal Susceptibility Testing

Cell suspensions for susceptibility testing (MMs and conventional antifungals) were prepared per the Clinical & Laboratory Standards Institute (CLSI) guidelines.^[^ ^54^, ^55^ ^]^ Before testing, yeasts (*C. albicans* and *S. cerevisiae*) were sub‐cultured in Sabouraud Dextrose Agar‐Emmons Modification (SDAE) plates and grown for 24 h at 30 °C. Five independent colonies from 24‐h‐old plates were collected and diluted to ≈10^4^ colony‐forming units (CFU) per mL in sterile saline.^[^ ^54^ ^]^ Molds (*A. fumigatus* and the dermatophytes *T. rubrum* and *M. gypseum*) were sub‐cultured on SDAE medium and incubated for 7 days at 28 °C. Conidia were recovered by covering the plates with sterile distilled water and scraping the colonies. The suspensions were filtered (8‐µm pore size) and diluted in saline to ≈10^4^ CFU mL^−1^.^[^ ^56^ ^]^

For MM MIC determination, increasing concentrations (0.3125–160 µm) of different MMs (8 mm stock in DMSO) were added to the cell suspensions. After a 30‐min incubation in the dark, cell suspensions were transferred to small, sterilized glass beakers, which were then placed in a water bath. Each sample was irradiated with a 405‐nm light at 292 mW cm^−2^ for 5 min, corresponding to a light dose of 87.6 J cm^−2^, determined using an S415C thermal power sensor (Thorlabs, Newton, MA, USA). During irradiation, the cell suspensions were agitated with a small metal stirrer. A thermocouple probe (model SC‐TT‐K‐30‐36‐PP; Omega Engineering, Inc., Stanford, CT, USA) was used to monitor the temperature during irradiation. Irradiated cell suspensions were inoculated in 3‐(*N*‐morpholino)propanesulfonic acid (MOPS)‐buffered Roswell Park Memorial Institute Medium (RPMI) 1640 (pH 7). Tubes were incubated at 30 °C for 48 h (yeasts) and 28 °C for 7 days (molds). The antifungal or MM concentration resulting in no visible growth was defined as MIC.^[^ ^54^, ^55^ ^]^ Similarly prepared cell suspensions were used to determine the MIC of conventional antifungals.

Aliquots (100 µL) of MIC tubes without visible fungal growth were plated on the SDAE medium. Plates were incubated at 30 °C for 48 h with confirmation after 72 h (yeasts) and for 7 days at 28 °C with confirmation after 14 days (molds). The lowest concentration that killed ≥99.9% of the original inoculum was defined as MFC.^[^ ^17^ ^]^

### Time‐Kill Assays

For yeasts, five independent colonies were collected from 24‐h SDAE plates, inoculated into yeast peptone with 2% dextrose (YPD), and grown for 24 h at 30 °C. Cells were then sub‐cultured in fresh medium and grown for ≈9 h. Afterward, the cells were centrifuged (5000 × g, 5 min), washed, and resuspended in phosphate‐buffered saline (PBS) to ≈10^6^ CFU mL^−1^. For *A. fumigatus*, conidia suspensions (≈10^4^ CFU mL^−1^) were prepared in PBS as previously described.

Cell/conidia suspensions were treated with 1% DMSO or MMs (2× MIC), and after a 30‐min dark incubation, irradiated (405‐nm light at 292 mW cm^−2^) as previously described. Similarly processed samples treated with a slow MM (10 µm, corresponding to the maximum MM MIC detected across all fungal strains) served as a control for the effects of MM rotation speed on antifungal activity. AMB (4 × MIC) controls were prepared likewise, but the light was omitted. Aliquots were collected in 1‐min increments for up to 10 min, serially diluted in PBS, and plated on SDAE medium. Plates were incubated at 30 °C for 48 h with confirmation after 72 h (yeasts) or at 28 °C for 7 days with confirmation after 14 days (*A. fumigatus*), after which the CFU number was determined. The results were expressed as the logarithm of base 10 of the ratio between the CFU at each time point and the CFU at time 0. The detection limit of the method was ≈1 log_10_ CFU mL^−1^.

### Biofilm Viability and Biomass

The antibiofilm activity of MMs was investigated using 96‐well microtiter plates with flat‐bottom wells as a closed static biofilm reactor. This setup is reliable, inexpensive, easy to use and obtain, and requires no additional equipment.^[^ ^57^ ^]^ Two parameters were used to evaluate antibiofilm activity: biofilm biomass and biofilm viability. Biofilm biomass was determined using the crystal violet method,^[^ ^19^ ^]^ a simple, inexpensive, and readily accessible method for determining biofilm biomass. However, because crystal violet binds both live and dead cells, as well as extracellular polymeric substances, it cannot be used alone to reliably assess antibiofilm activity. To overcome this limitation, the XTT assay was used to evaluate biofilm viability.^[^ ^18^ ^]^ This assay was based on the reduction of the tetrazolium salt XTT to formazan by dehydrogenases in the mitochondrial electron transport chain of living cells. The resulting formazan can be easily detected by measuring the absorbance at 490 nm, which was proportional to the number of living cells, providing a reliable quantitative measurement of metabolically active cells in biofilms.^[^ ^58^ ^]^

*C. albicans* biofilms were established in 96‐well flat‐bottom polystyrene plates (Corning‐Costar Corp., Corning, NY, USA) by diluting 24‐h cultures in fresh MOPS‐buffered RPMI 1640. After 48 h at 30 °C, mature biofilms were washed with PBS and treated with AMB (2 × or 4 × MIC), 1% DMSO, or different MMs (2 × or 4 × MIC). DMSO‐ and MM‐treated samples were then irradiated in situ with 405‐nm light (87.6 J cm^−2^).

Biofilm viability was determined using an XTT cell viability assay kit (Biotium, Hayward, CA, USA) per the manufacturer's instructions. Absorbance (490 nm) and background (640 nm) were read in a microplate reader (BioTek Instruments Inc., Winooski, VT, USA). Normalized absorbance values were obtained by subtracting the background from the signal.

Biofilm biomass was determined by the crystal violet method, as previously described.^[^ ^19^ ^]^ The absorbance of the supernatant at 550 nm was determined in a microplate reader (BioTek Instruments Inc., Winooski, VT, USA). Untreated sample values minus background were defined as 100% and used to calculate biofilm viability and biomass reduction after treatment.

### Development of Resistance to Visible‐Light‐Activated MMs

A modified version of the broth macrodilution serial passage method was used to assess the development of resistance to visible‐light‐activated MMs in *C. albicans*.^[^ ^59^ ^]^ *C. albicans* cell suspensions were prepared and irradiated as previously described for the determination of MM MIC. Cells were then inoculated into buffered RPMI 1640 and incubated at 30 °C for 48 h. Cells able to grow at 0.5× MIC of MM were centrifuged (5000 × *g*, 5 min), resuspended, rechallenged with different MM concentrations, and irradiated with 405‐nm light (87.6 J cm^−2^). The procedure was repeated for 20 consecutive cycles. The antifungals AMB, CAS, and FLC were processed similarly, except that light was omitted, and used as control.

### Plasma Membrane Permeability

The effects of MMs on plasma membrane permeability were determined by monitoring PI uptake^[^ ^60^ ^]^ and calcein leakage.^[^ ^61^ ^]^

For PI uptake, *C. albicans* cells were grown as described for time‐kill experiments, centrifuged (5000 × *g*, 5 min), washed, and resuspended in 5 mm glucose and 5 mm 4‐(2‐hydroxyethyl)‐1‐piperazineethanesulfonic acid (HEPES) buffer (pH 7.2). Cell suspensions (≈10^6^ CFU mL^−1^) were treated with 1% DMSO or visible‐light‐activated MMs (0.5–2× MIC) and then irradiated with 405‐nm light (87.6 J cm^−2^). After irradiation, PI (10 µm final concentration) was added to the cells. PI‐labeled cells were transferred to a black 96‐well plate, and PI fluorescence (excitation: 535 nm, emission: 617 nm) over time was monitored in a microplate reader (BioTek Instruments Inc., Winooski, VT, USA).

For calcein leakage assays, *C. albicans* cells (≈10^6^ CFU mL^−1^), grown as described for time‐kill experiments, were centrifuged (5000 × g, 5 min), washed, and resuspended in assay buffer (20 mm MOPS sodium salt, 1 mm CoCl_2_, 90 mm NaCl, pH 7.5) containing 0.8 mm calcein‐AM. After a 2‐h incubation at 30 °C, calcein‐loaded cells were diluted (≈10^5^ CFU mL^−1^) in assay buffer, treated with MMs (0.5–2× MIC) or 1% DMSO and irradiated with 405‐nm light (87.6 J cm^−2^). Afterward, the cells were centrifuged (5000 × *g*, 5 min) and resuspended in assay buffer. At least 10 000 cells were then analyzed in a Sony SA3800 spectral analyzer (Sony Biotechnology, CA, USA).

### Intracellular and Extracellular ATP

*C. albicans* cell suspensions (≈10^6^ CFU mL^−1^) were treated with 1% DMSO or MMs (0.5–2 × MIC) and irradiated with 405‐nm light (87.6 J cm^−2^), as described above. Following centrifugation (5000 × *g*, 5 min), extracellular and intracellular ATP were extracted from the supernatant and pellet, respectively, as previously described.^[^ ^62^ ^]^

ATP concentrations were measured using the CellTiter‐Glo Luminescent Cell Viability Assay (Promega, Madison, WI, USA) per the manufacturer's instructions. The luminescent signal was measured using a microplate reader (BioTek Instruments Inc., Winooski, VT, USA) and converted to ATP concentration by linear regression of a standard ATP curve prepared using adenosine 5′‐triphosphate disodium salt trihydrate. ATP levels were normalized to the protein concentration determined using the Pierce Assay (Pierce BCA Protein Assay Kit, Thermo Fisher Scientific, MA, USA).

### Plasma Membrane Fluidity

The effects of MMs on *C. albicans* membrane dynamics were evaluated using DPH fluorescence.^[^ ^20^ ^]^ *C. albicans* cell suspensions (≈10^6^ CFU mL^−1^) were prepared, treated with 1% DMSO or MMs (0.5–2 × MIC), and then irradiated with 405‐nm light (87.6 J cm^−2^). AMB‐treated cells were used as controls. Samples were fixed with 0.37% formaldehyde and labeled with 0.6 mm DPH, as previously described.^[^ ^20^ ^]^ DPH fluorescence (excitation: 350 nm, emission: 420 nm) was measured in a microplate reader (BioTek Instruments Inc., Winooski, VT, USA). DPH fluorescence of untreated samples minus background was defined as 100% and used to calculate changes in treated samples.

### Competition Assays with Exogenous Ergosterol and Phospholipids

Competition assays with exogenous ergosterol and phospholipids were performed as previously described^[^ ^63^ ^]^ with modifications. *C. albicans* cell suspensions (≈10^6^ CFU mL^−1^) were prepared as described for time‐kill assays to which increasing concentrations (up to 100 µg mL^−1^) of exogenous ergosterol or the phospholipids phosphatidylcholine, phosphatidylethanolamine, phosphatidylglycerol, or cardiolipin (Avanti Polar Lipids, AL, USA) were added. Increasing concentrations of MM were then added to each ergosterol‐ and phospholipid‐treated sample. After a 30‐min dark incubation, the samples were irradiated with 405‐nm light (87.6 J cm^−2^) as previously described. Buffered RPMI 1640 medium was then added to the irradiated samples. After incubation at 30 °C for 48 h, samples were examined for growth to determine the MM MIC.

### Electron Microscopy

*C. albicans* cell suspensions (≈10^6^ CFU mL^−1^) were prepared in PBS (1×) as described for time‐kill assays, treated with 1% DMSO or 0.5 × MIC MM **1**, and then irradiated with 87.6 J cm^−2^ 405‐nm light. Irradiated cells were fixed with Karnovsky's fixative, postfixed with 1% osmium, and dehydrated with a series of ethanol washes. For TEM, specimens were embedded in epoxy resin (PolyBed 812; Polysciences, Inc., Warrington, PA, USA) after being dehydrated in a series of washes with a graded concentration of 50–100% ethanol. A Leica EM UC7 ultramicrotome (Leica Microsystems, Wetzlar, Germany) was used to cut ultrathin sections (65 nm), which were then poststained with uranyl acetate and lead citrate. Samples were observed using a JEOL JEM2100 TEM (Hitachi Corporation, Japan) operating at an accelerating voltage of 80 kV. For SEM, after dehydration with ethanol, samples were dried with a Leica EM CPD300 (Leica Microsystems, Wetzlar, Germany) at the critical point, sputter‐coated with 10 nm gold, and imaged with an FEI Apreo SEM (FEI Apreo, ThermoFisher Scientific, Waltham, MA, USA) using a secondary electron detector.

### Colocalization Analysis

Colocalization analysis of MMs was performed as previously described^[^ ^64^, ^65^ ^]^ with modifications. A single isolated colony was picked from 24‐h SDAE plates, diluted in liquid YPD, and grown at 30 °C for 24 h. Cells were then re‐diluted in fresh YPD medium and grown statically in Ibidi µ‐dishes (Ibidi GmbH, Munich, Germany) for 24 h at 30 °C. The cells were washed, and then YPD medium containing 8 µm MM **1** and 10 nm MitoTracker Green (Thermo Fisher Scientific, MA, USA) was added. After a 30‐min dark incubation at 30 °C, the solution was replaced with fresh medium containing 40 nM FM 4–64 (Thermo Fisher Scientific, MA, USA). Cells were immediately imaged in a Nikon A1‐RSI confocal system mounted on a Nikon Ti‐E widefield fluorescence microscope (Nikon Corporation, NY, USA). Cells were imaged directly on the Ibidi imaging dish using a 60 × water immersion objective (numerical aperture of 1.27, 0.17 mm working distance). Colocalization was calculated in the Fiji version of ImageJ using the Colocalization Threshold tool and the Coloc‐2 plugin.

### Mitochondrial Activity

The effect of visible‐light‐activated MMs on mitochondrial activity was assessed using XTT, which was metabolically reduced by mitochondrial dehydrogenases.^[^ ^66^ ^]^

*C. albicans* cell suspensions (≈10^6^ CFU mL^−1^), prepared as described for time‐kill experiments, were treated with 1% DMSO or MMs (0.5–2 × MIC) and irradiated with 405‐nm light (87.6 J cm^−2^). Irradiated cells were mixed with 25 µL of activated XTT working solution (Biotium, Hayward, CA, USA) in a 96‐well plate. After 4 h at 30 °C, the absorbance (490 nm) and background (640 nm) were measured in a microplate reader (BioTek Instruments Inc., Winooski, VT, USA). The absorbance of untreated samples minus background was defined as 100% and used to calculate the reduction in mitochondrial activity.

### Mitochondrial ROS

*C. albicans* cell suspensions (≈10^6^ CFU mL^−1^) prepared as described above were treated with 1% DMSO or MMs (0.5–2 × MIC) and then irradiated with 405‐nm light (87.6 J cm^−2^). Afterward, the cells were centrifuged (5000 × *g*, 5 min), washed, and resuspended in PBS (≈10^6^ cells mL^−1^). Mitochondrial ROS was quantified using the fluorescent superoxide radical‐sensitive probe MitoROS 580 (AAT Bioquest, CA, USA) per the distributor's instructions. The fluorescence of MitoROS 580 (excitation: 510 nm, emission: 580 nm) over time was monitored in a microplate reader (BioTek Instruments Inc., Winooski, VT, USA).

Mitochondrial ROS generation was also monitored by confocal microscopy. Cells were prepared as previously described for colocalization analysis and then mixed with an equal volume of 2× MitoROS 580 working solution in Hank's Balanced Salt Solution with 20 mm HEPES (HHBS) buffer containing 1.25 µm MM **1**. After a 30‐min dark incubation, the solution was removed and replaced with fresh HHBS buffer. Cells were immediately imaged under a Nikon A1 confocal microscope (Nikon Corporation, NY, USA) as previously described. MM light activation was performed in situ with a SOLA LED using a DAPI excitation filter (395/25 nm, 166 mW cm^−2^) for 5 min. Fluorescence intensities were extracted from microscopy images using FIJI's built‐in algorithms.

### Superoxide Dismutase Activity and Lipid Peroxidation

*C. albicans* cell suspensions (≈10^6^ CFU mL^−1^) were prepared as described above, challenged with 1% DMSO or MMs (0.5–2 × MIC), and then irradiated with 405‐nm light (87.6 J cm^−2^), after which the cells were centrifuged (5000 × *g*, 5 min). SOD activity was determined using a SOD Assay Kit (Caymanchem, MI, USA) per the distributor's instructions. Lipid peroxidation was determined using a Thiobarbituric Acid Reactive Substances (TBARS) assay kit (TCA method) (Caymanchem, MI, USA) per the distributor's instructions. SOD activity and malondialdehyde (MDA) levels were normalized by protein content determined by the Pierce assay (Pierce BCA Protein Assay Kit, Thermo Fisher Scientific, MA, USA).

### Mitochondrial Membrane Potential

Changes in mitochondrial membrane potential were determined by monitoring the fluorescence shift of the ratiometric mitochondrial membrane potential probe JC‐1.^[^ ^67^ ^]^ *C. albicans* cell suspensions (≈10^6^ CFU mL^−1^) were treated with DMSO or MMs (0.5–2 × MIC), irradiated with 405‐nm light (87.6 J cm^−2^), and then labeled with 5 µm JC‐1 (ABP Biosciences, MD, USA) per the distributor's instructions. At least 10 000 cells per sample were then analyzed in an SA3800 Spectral Analyzer (Sony Biotechnology, CA, USA).

### Intracellular Calcium Levels

Calcium levels were measured using the fluorescent probes Calbryte 520 AM (AAT Bioquest, CA, USA) and Rhod‐2 AM (AAT Bioquest, CA, USA) to determine cytosolic and mitochondrial calcium levels, respectively.^[^ ^68^, ^69^ ^]^ *C. albicans* cell suspensions (≈10^6^ CFU mL^−1^) were prepared in HHBS containing 0.04% Pluronic F‐127 (AAT Bioquest, CA, USA) and labeled with Rhod‐2 AM or Calbryte 520 AM (4 µm final concentration). After a 30‐min dark incubation at 30 °C, 1% DMSO or MMs (0.5–2 × MIC) was added. Following an additional 30‐min incubation, the cells were centrifuged (5000 × *g*, 5 min), resuspended in HHBS, and irradiated with 405‐nm light (87.6 J cm^−2^). Afterward, the cells were centrifuged (5000 × *g*, 3 min) and resuspended in HHBS. The fluorescence of Calbryte 520 AM (excitation = 490 nm, emission = 525 nm) and Rhod‐2 AM (excitation = 540 nm, emission = 590 nm) over time was monitored in a microplate reader (BioTek Instruments Inc., Winooski, VT, USA) or by flow cytometry in an SA3800 Spectral Analyzer (Sony Biotechnology, CA, USA).

Calcium levels were also monitored by live‐cell calcium imaging using confocal microscopy. Cells were grown as described for colocalization experiments. The growth medium was then replaced with fresh HHBS buffer containing Rhod‐2 AM (4 µm final concentration), to which MM **1** (1.25 µm) was added. After a 30‐min dark incubation, the solution was replaced with fresh HHBS. Cells were immediately imaged using a Nikon A1 confocal microscope (Nikon Corporation, NY, USA) directly on the Ibidi imaging dish with a 60× water immersion objective. MM light activation was performed in situ with a SOLA LED using a DAPI excitation filter (395/25 nm, 166 mW cm^−2^). Light was delivered through the microscope objective for 5 min, after which fluorescence was monitored for 60 additional minutes. Fluorescence intensities were extracted from microscopy images using FIJI's built‐in algorithms.

### Influence of BAPTA‐AM on MM‐Induced Killing and Intracellular Calcium Levels

*C. albicans* cells were grown as described above and resuspended in HHBS (≈10^6^ CFU mL^−1^). The cation chelator 1,2‐bis(2‐aminophenoxy)ethane‐*N,N,N',N′*‐tetraacetic acid acetoxymethyl ester (BAPTA‐AM) was then added (0.25–1 mm, final concentration).^[^ ^38^ ^]^ Unamended cells were used as controls. After a 30‐min dark incubation at 30 °C, the cells were centrifuged (5000 × *g*, 5 min), washed, and resuspended in HHBS. MM **1** (2× MIC) was then added. After 30 min, the cells were irradiated and processed as described for the time‐kill experiments.

Intracellular calcium levels in untreated cells or cells treated with BAPTA‐AM (1 mm) and then treated with 1% DMSO or different concentrations of MMs (0.5–2 × MIC) plus 405‐nm light (87.6 J cm^−2^) were determined using the probes Calbryte 520 AM and Rhod‐2 AM, as described above.

### Mitochondrial Mass/Volume

Mitochondrial mass/volume was estimated using MitoTracker Green fluorescence.^[^ ^70^ ^]^ *C. albicans* cell suspensions (≈10^6^ CFU mL^−1^) were treated with DMSO or MMs (0.5–2 × MIC) and then irradiated with 405‐nm light (87.6 J cm^−2^). The cells were then stained with MitoTracker Green (200 nm) for 30 min at 30 °C and washed three times with PBS. At least 10 000 cells per sample were analyzed in an SA3800 Spectral Analyzer (Sony Biotechnology, CA, USA).

### Cytochrome c Release

*C. albicans* cell suspensions (≈10^6^ CFU mL^−1^) were treated with DMSO or MMs (0.5–2× MIC) and irradiated with 405‐nm light (87.6 J cm^−2^). Cells were harvested for protoplast preparation by digestion with zymolyase 20 T (20 mg mL^−1^, US Biological Life Sciences, MA, USA) in 0.1 m potassium phosphate buffer (pH 6) containing 1 m sorbitol for 1 h at 30 °C. Mitochondrial cytochrome c was extracted and reduced with ascorbic acid (0.5 mg mL^−1^) as previously described.^[^ ^71^ ^]^ The absorbance at 550 nm was determined on a Beckman Coulter DU 800 spectrophotometer (Fullerton, CA, USA). Cytochrome c levels were normalized to the protein content determined using the Pierce assay (Pierce BCA Protein Assay Kit, Thermo Fisher Scientific, MA, USA).

### Detection of Necrosis and Apoptosis

The occurrence of necrosis and apoptosis was investigated using an Annexin V‐FITC/PI assay.^[^ ^24^ ^]^ *C. albicans* cells were grown as described for time‐kill experiments, washed in sorbitol buffer (0.5 mm MgCl_2_, 35 mm potassium phosphate, pH 6.8, containing 1.2 m sorbitol), and resuspended in the same buffer containing zymolyase 20 T (20 mg mL^−1^, US Biological Life Sciences, MA, USA). After 1 h of digestion at 30 °C, protoplasts were centrifuged, washed, and resuspended in binding buffer (140 mm NaCl, 10 mm HEPES, 2.5 mm CaCl_2_, 1.2 m sorbitol, pH 7.4). Protoplasts were treated with 1% DMSO or MMs (0.5–2 × MIC) and then irradiated with 405‐nm light (87.6 J cm^−2^). The protoplasts were immediately labeled using an Annexin V‐FITC/PI Apoptosis Kit (Abnova, Taiwan) per the distributors' instructions. At least 10 000 cells per sample were analyzed in an SA3800 spectral analyzer (Sony Biotechnology, CA, USA).

### Interaction between Visible‐Light‐Activated MMs and Conventional Antifungals

The interaction of MMs with conventional antifungal agents in *C. albicans* was investigated by determining the MIC of different antifungals alone and after treatment with visible‐light‐activated MMs using a modified broth microdilution checkerboard assay^[^ ^72^ ^]^ in an 8 × 8‐well configuration*. C. albicans* cell suspensions were prepared as described for MIC determination and treated with increasing concentrations (up to 1× MIC) of MMs. Following irradiation (87.6 J cm^−2^ of 405‐nm light), cells were collected and distributed along the *x*‐axis of a 96‐well plate. Increasing concentrations (up to 1× MIC) of different antifungal drugs (Table S2, Supporting Information) in geometric twofold increments in buffered RPMI 1640 medium were added along the plate's *y*‐axis. After 48 h at 30 °C, the absorbance at 630 nm was measured in a microplate reader (BioTek Instruments Inc., Winooski, VT, USA). The FICI was determined as the sum of the MIC of the MM and the antifungal drug when used in combination and divided by their MIC when used alone. A FICI index of ≤ 0.5, 0.5 < *x* ≤ 4, or > 4 indicated synergistic, additive, and antagonistic interactions, respectively.^[^ ^25^ ^]^

### Efflux Activity

Efflux pump activity was evaluated by measuring the energy‐dependent efflux of the fluorescent dye rhodamine 6G.^[^ ^73^ ^]^ *C. albicans* cells were grown overnight (≈16 h) in YPD at 30 °C, rediluted in fresh YPD, and grown for an additional 3 h at 30 °C. The cells were then centrifuged, washed with 50 mm HEPES buffer (pH 7), and resuspended in de‐energization buffer containing 1 µm antimycin A and 5 mm 2‐deoxy‐D‐glucose in 50 mm HEPES buffer (pH 7). After 3 h at 30 °C, the cells were centrifuged, washed, and resuspended in cold 50 mm HEPES buffer (pH 7). The cells were then incubated with rhodamine 6G (10 µm final concentration) for 2 h at 30 °C. Afterward, the cells were centrifuged (1000 × *g*, 5 min), washed, and resuspended in cold HEPES buffer. Cells were then treated with 1% DMSO or MMs (0.5–2× MIC) and irradiated with 405‐nm light (87.6 J cm^−2^). Irradiated cells were collected and incubated in prewarmed HEPES buffer containing 1 mm glucose for 1 h at 30 °C to reactivate the cells. Afterward, the cells were centrifuged (1000 × *g*, 5 min), resuspended in HEPES buffer, and transferred to a 96‐well plate. Rhodamine 6G fluorescence (excitation: 485 nm, emission: 535 nm) over time was measured in a microplate reader (BioTek Instruments Inc., Winooski, VT, USA). Rhodamine 6G‐free cells served as unstained controls. Untreated sample fluorescence minus background was defined as 100% and used to normalize the remaining data points.

### Toxicity Profiling and Therapeutic Index Calculation

The biocompatibility of MMs with primary HEK293T cells was assessed using the CellTiter‐Glo Luminescent Cell Viability Assay (Promega, WI, USA) per the manufacturer's instructions by treating cells with increasing concentrations of different MMs plus 405‐nm light (87.6 J cm^−2^). Dose‐response curves were used to determine the MM concentrations that reduced cell viability by 50% (IC_50_). The therapeutic index was calculated as the ratio between the IC_50_ and the MIC.

### In Vivo Antifungal Activity of MMs

The in vivo antifungal activity of MMs was assessed in *G. mellonella*.^[^ ^47^, ^48^ ^]^ *G. mellonella* were acquired from a commercial supplier (rainbowmealworms.net) in their final instar larval stage. Worms of similar size (≈0.3 g), responsive to touch, and displaying no signs of melanization were selected. *C. albicans* (≈10^5^ CFU mL^−1^) and *A. fumigatus* conidia (≈10^4^ conidia mL^−1^) suspensions were prepared in PBS as previously described. The fungal inoculum (5 µL) was injected into the last left proleg of the worms with a Hamilton syringe. 30 min after infection, MM and/or antifungal agents (1× MIC, Table S2, Supporting Information) diluted in sterile water were injected similarly to the right proleg. The following treatment groups (eight individuals each, from three independent batches) were established: 1) 1% DMSO with and without light, 2) monotherapy with MM **1** alone (1× MIC) with and without light, 3) monotherapy with conventional antifungals (1× MIC) AMB or azole (FLC, in the case of *C. albicans* and VRC, in the case of *A. fumigatus*), or 4) combination therapy with visible‐light‐activated MM **1** (1× MIC) followed by treatment with a conventional antifungal (1× MIC). After 30 min, worms in the irradiated treatment groups were transferred to 24‐well plates (Corning‐Costar Corp., Corning, NY, USA) and irradiated with 405‐nm light (87.6 J cm^−2^). Worms were incubated in sterile Petri dishes at 30 °C in the dark. Live and dead worms were scored each day for 7 days. Melanized or unresponsive worms were considered dead.

The fungal load was assessed in a separate group of similarly treated worms 48 h after infection. Only healthy larvae (four worms per treatment group) with no melanization spots were used. After weight determination, worms were killed by freezing and homogenized using a tissue grinder (Fisherbrand, Fisher Scientific, Pittsburgh, PA, USA). For *C. albicans*‐infected worms, after homogenization in sterile PBS, serial dilutions were plated on YPD agar containing antibiotics (100 µg mL^−1^ ampicillin, 100 µg mL^−1^ streptomycin, and 45 µg mL^−1^ kanamycin).^[^ ^47^ ^]^ For *A. fumigatus*‐infected worms, after homogenization in sterile PBS containing gentamicin (25 µg mL^−1^) and chloramphenicol (400 µg mL^−1^), serial dilutions were plated on potato dextrose agar (PDA).^[^ ^74^ ^]^ After 48 h at 30 °C (*C. albicans*) or 7 days at 28 °C (*A. fumigatus*), colonies were counted to determine CFU per mg of larvae.

Work on *G. mellonella* was reviewed and approved by the Office of Sponsored Projects and Research Compliance (SPARC) at Rice University.

### Ex Vivo Model of Onychomycosis

For microconidia preparation, *T. rubrum* was inoculated on PDA containing 0.025% Sabouraud dextrose broth (SDB) and 1% penicillin‐streptomycin. After a 10‐day incubation at 28 °C, the plates were flooded with PBS, which was then aspirated and filtered through a sterilized cotton gauze to recover microconidia.^[^ ^75^ ^]^

An ex vivo onychomycosis model was established as previously described^[^ ^76^ ^]^ with modifications. Pig hooves with exposed toenails were processed into ≈1 cm^2^‐sized individual toenail samples with a band saw, washed with 70% ethyl alcohol and sterilized water, and inoculated with a microconidia suspension of *T. rubrum* (≈10^7^ conidia mL^−1^) for 3 h. Samples were placed in a Petri dish containing moist sterilized paper and incubated at 28 °C for 10 days. Fungal growth was confirmed by sample resuspension in PBS and plating on PDA containing 0.025% SDB and 1% penicillin‐streptomycin. Infected samples were then treated with 1) 1% DMSO plus light, 2) monotherapy with MM **1** alone (0.77% in DMSO) plus light, 3) monotherapy with a conventional antifungal (three drops^[^ ^76^ ^]^ of Ciclopirox Topical Suspension USP, 0.77% “Lotion,” Leading Pharma, LLC, NY, USA, or Ciclopirox Topical Solution, 8% “Lacquer,” Perrigo New York Inc., NY, USA), or 4) combination therapy with MM **1** plus light and conventional antifungal. Each treatment group consisted of three samples. After 30 min, samples in the irradiated treatment groups were transferred to 24‐well plates (Corning‐Costar Corp., Corning, NY, USA) and irradiated with 405‐nm light (87.6 J cm^−2^). Treatment was repeated every 24 h for 5 days. Afterward, the samples were transferred to tubes containing PBS plus 1% penicillin‐streptomycin, vortexed, and sonicated.^[^ ^76^ ^]^ Triplicate aliquots of this suspension were inoculated on PDA plates containing 1% penicillin‐streptomycin. After a 10‐day incubation at 28 °C, CFU numbers were determined. Untreated samples served as positive controls.

### Statistical Analysis

Unless otherwise noted, all experiments were performed at least in triplicate. The arithmetic mean and the standard deviation or the standard error of the mean across biological and technical replicates were used as measures of mean and spread. No data points were excluded as outliers. When appropriate, data were normalized to a 0–100% range. All data processing and statistical analyses were performed using GraphPad Prism 8.0 (San Diego, CA, USA). Depending on the sample size, the normality of the data was assessed using an Anderson–Darling normality test, a D'Agostino‐Pearson omnibus normality test, a Shapiro–Wilk normality test, or a Kolmogorov–Smirnov normality test with the Dallal–Wilkinson–Lilliefors test for *p* values. Comparisons between the two groups were performed with a *t*‐test for parametric data or a Mann–Whitney U test for nonparametric data. Comparisons between multiple groups were performed using ANOVA or a Kruskal–Wallis test with Dunn's multiple comparisons test. A Mantel–Cox test was used to determine statistical significance in *G. mellonella* survival experiments. Unless otherwise stated, all figures were generated in GraphPad Prism 8.0 (San Diego, CA, USA). Flow cytometry data were initially analyzed and visualized in FlowJo software (version 9, Tree Star Inc., Ashland, OR, USA) and exported to GraphPad for statistical analysis. A value of *p* <0.05 was considered statistically significant. Asterisks are used where appropriate to indicate the significance of differences. *p <0.05, **p <0.01, ***p <0.001, ****p <0.0001. Confocal microscopy images were processed and analyzed using the appropriate plugins in Fiji/ImageJ (National Institutes of Health, MD, USA).

## Conflict of Interest

Rice University owns intellectual property on the use of electromagnetic (light) activation of MMs for the killing of cells. This intellectual property has been licensed to a company in which J.M.T. is a stockholder, although he is not an officer or director of that company. Conflicts of interest are mitigated through regular disclosure to the Rice University Office of Sponsored Projects and Research Compliance. The remaining authors declare no conflict of interest.

## Supporting information

Supporting Information

## Data Availability

The data that support the findings of this study are available from the corresponding author upon reasonable request.
