# Supplementary material for: Visible‐Light‐Activated Molecular Machines Kill Fungi by Necrosis Following Mitochondrial Dysfunction and Calcium Overload
Source: Adv Sci (Weinh). 2023 Jan 30;10(10):2205781. doi: 10.1002/advs.202205781 (PMC10074111; doi:10.1002/advs.202205781)
Supplement: Supplementary file 1 — Supporting Information [file ADVS-10-2205781-s001.pdf]

ADVANCED SCIENCE
Open Access

Supporting Information

for *Adv. Sci.*, DOI 10.1002/advs.202205781

Visible-Light-Activated Molecular Machines Kill Fungi by Necrosis Following Mitochondrial Dysfunction and Calcium Overload

Ana L. Santos, Jacob L. Beckham, Dongdong Liu, Gang Li, Alexis van Venrooy, Antonio Oliver, George P. Tegos and James M. Tour**

Supporting Information for

Visible-Light-Activated Molecular Machines Kill Fungi by Necrosis Following Mitochondrial Dysfunction and Calcium Overload

Ana L. Santos*, Jacob L. Beckham, Dongdong Liu, Gang Li, Alexis van Venrooy, Antonio Oliver, George P. Tegos, James M. Tour*

*Lead corresponding author: tour@rice.edu

Correspondence: alsantos@ua.pt, tour@rice.edu

Contents

Supporting Tables 2

Supporting Figures 7

Supplementary Materials and Methods 26

Supporting Tables

Table S1. Chemical structures of the visible-light-activated MMs investigated in this study, their corresponding molecular weights, and their estimated rotation rates after light activation calculated based on the rotation rates of known motors with similar core skeletons [1]. A slow MM (ARV-3-262) was used to assess the importance of fast rotational speeds for the antifungal activity of MMs. Detailed information on the synthesis and characterization of the piperazine-modified motor MM **7** can be found in the supplemental materials. For the remaining molecules, detailed information on synthesis and characterization can be found elsewhere [2]. Chemical structures were created in Chemdraw Ultra 6.0 (CambridgeSoft, Cambridge, MA, USA).

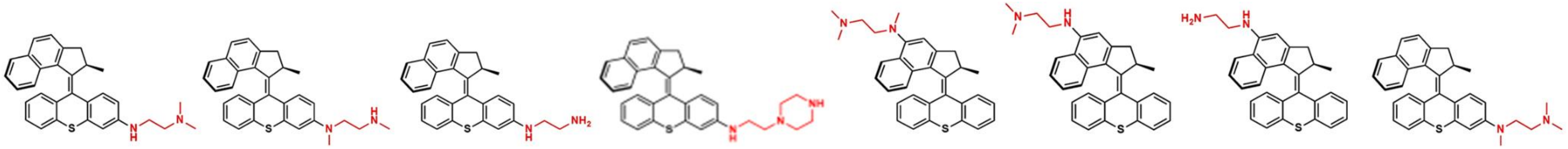

MM 1 (DL-4-654)	MM 5 (DL-5-877)	MM 6 (DL-5-878)	MM 7 (GL-82)	DL-4-751	DL-4-726	DL-5-873	DL-5-876
MW = 462.6550	MW = 462.6550	MW = 434.6010	MW = 503.2395	MW = 476.682	MW = 462.6550	MW = 434.6010	MW = 476.6820
3 MHz	3 MHz	3 MHz	3 MHz	3 MHz	3 MHz	3 MHz	3 MHz

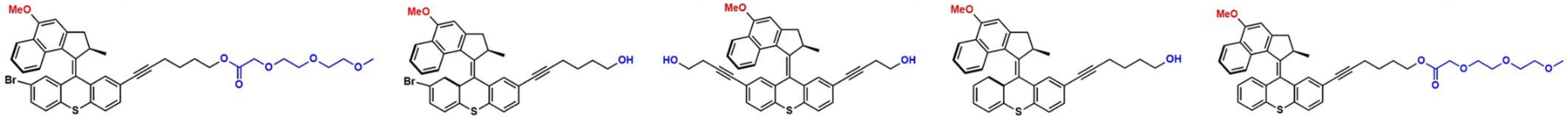

DL-4-655	DL-4-653	DL-4-713	DL-4-781	DL-4-795
MW = 662.8410	MW = 485.6650	MW = 642.6930	MW = 504.6880	MW = 662.8410
3 MHz	3 MHz	3 MHz	3 MHz	3 MHz

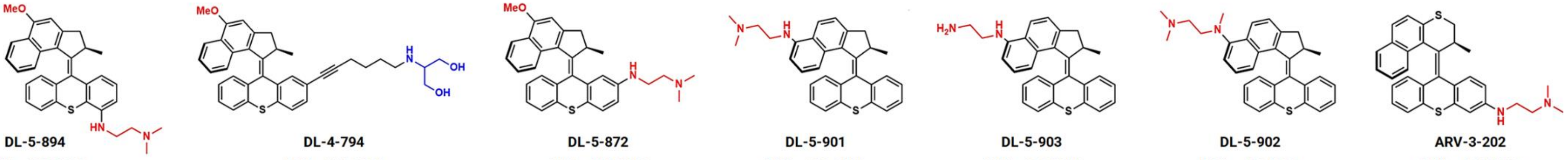

DL-5-894	DL-4-794	DL-5-872	DL-5-901	DL-5-903	DL-5-902	ARV-3-202
MW = 492.6810	MW = 575.2494	MW = 492.6810	MW = 462.6550	MW = 434.6010	MW = 476.6820	MW = 494.7150
3 MHz	3 MHz	3 MHz	3 MHz	3 MHz	3 MHz	10^{-6} Hz

Table S2. Minimum inhibitory concentration (MIC, µg ml^{-1}) of various antifungal agents in different fungal strains used in this study determined using the broth microdilution method according to the CLSI guidelines for yeasts (CLSI M27-A2) [3] and molds (CLSI M38-A2) [4]. Further details are provided in the main text. The results are the average of at least three independent biological replicates.

	C. albicans	*A. fumigatus*	*S. cerevisiae*	*T. rubrum*
**5-Fluorocytosine (5-FC)**	1	-	-	-
**Amphotericin B (AMB)**	1	2	0.5	-
**Fluconazole (FLC)**	0.5	-	-	-
**Voriconazole (VRC)**	0.03	0.5	0.125	-
**Caspofungin (CAS)**	0.25	-	0.5	-
**Ciclopirox (CPX)**	1	-	-	0.5

Table S3. Susceptibility (assessed as the MIC in µM) of antifungal-resistant (^{R}) *C. albicans* to MMs. During serial passage experiments used to assess antifungal resistance, cells that grew at 0.5× MIC for each antifungal were collected and stored at -80 °C. These cells were then re-grown, amended with a range of MM concentrations and irradiated with 405 nm light (87.6 J cm^{-2}). Subsequently, the irradiated cells were inoculated into MOPS-buffered RPMI 1640 media and grown at 30 °C for 48 h. Further experimental details can be found in the main text. The tubes were then inspected for growth to determine the MIC. The results are the mean of at least three biological replicates. FLC: Fluconazole. CAS: Caspofungin.

	FLC^{R}				CAS^{R}			
Passage number	MM **1**	MM **5**	MM **6**	MM **7**	MM **1**	MM **5**	MM **6**	MM **7**
1	1.25	2.5	2.5	5	1.25	2.5	2.5	5
2	1.25	2.5	2.5	5	1.25	2.5	2.5	5
3	1.25	2.5	2.5	5	1.25	2.5	2.5	5
4	1.25	2.5	2.5	5	1.25	2.5	2.5	5
5	1.25	2.5	2.5	5	1.25	2.5	2.5	5
6	1.25	2.5	2.5	5	1.25	2.5	2.5	5
7	1.25	2.5	2.5	5	1.25	2.5	2.5	5
8	1.25	2.5	2.5	5	1.25	2.5	2.5	5
9	1.25	2.5	2.5	5	1.25	2.5	2.5	5
10	1.25	2.5	2.5	5	1.25	2.5	2.5	5
11	1.25	2.5	2.5	5	1.25	2.5	2.5	5
12	1.25	2.5	2.5	5	1.25	2.5	2.5	5
13	1.25	2.5	2.5	5	1.25	2.5	2.5	5
14	1.25	2.5	2.5	5	1.25	2.5	2.5	5
15	1.25	2.5	2.5	5	1.25	2.5	2.5	5
16	1.25	2.5	2.5	5	1.25	2.5	2.5	5
17	1.25	2.5	2.5	5	1.25	2.5	2.5	5
18	1.25	2.5	2.5	5	1.25	2.5	2.5	5
19	1.25	2.5	2.5	5	1.25	2.5	2.5	5
20	1.25	2.5	2.5	5	1.25	2.5	2.5	5

Table S4. Statistical comparison of survival curves of worms infected with *C. albicans* and treated with DMSO, MM **1**, antifungal or a combination of MM **1** and antifungal using the log-rank (Mantel–Cox) test in GraphPad Prism 8.0 (San Diego, CA, USA). ns: non-significant.

	1% DMSO (Dark)	1% DMSO	MM **1** (No Light)	MM **1**	Amphotericin B	Azole	MM **1** + Amphotericin B	MM **1** + Fluconazole
1% DMSO (Dark)	-	ns	ns	0.0022	0.0232	0.0002	<0.0001	0.0009
1% DMSO	-	-	0.0324	0.001	0.0123	<0.0001	<0.0001	0.0032
MM **1** (Dark)	-	-	-	0.002	0.0198	0.0002	<0.0001	0.0118
MM **1**	-	-	-	-	ns	0.0152	0.0001	ns
Amphotericin B	-	-	-	-	-	0.0114	0.0001	0.0440
Fluconazole	-	-	-	-	-	-	0.0002	ns
MM **1**+ Amphotericin B	-	-	-	-	-	-	-	0.0122

Table S5. Statistical comparison of survival curves of worms infected with *A. fumigatus* and treated with DMSO, MM **1**, antifungal or a combination of MM **1** and antifungal using the log-rank (Mantel–Cox) test in GraphPad Prism 8.0 (San Diego, CA, USA). ns: non-significant.

	1% DMSO (Dark)	1% DMSO	MM **1** (Dark)	MM **1**	Amphotericin B	Voriconazole	MM **1** + Amphotericin B	MM **1** + Voriconazole
1% DMSO (Dark)	-	ns	ns	ns	ns	ns	0.0096	0.006
1% DMSO	-	-	ns	ns	ns	ns	0.0407	0.0278
MM **1** (Dark)	-	-	-	ns	ns	ns	0.0135	0.0067
MM **1**	-	-	-	-	ns	ns	ns	ns
Amphotericin B	-	-	-	-	-	ns	ns	ns
Voriconazole	-	-	-	-	-	-	ns	ns
MM **1** + Amphotericin B	-	-	-	-	-	-	-	ns

Table S6. Statistical comparison of fungal load in porcine nails infected with *T. rubrum* and treated with DMSO plus 405 nm light at 87.6 J cm^{-2}, visible-light-activated MM **1** plus 405 nm light at 87.6 J cm^{-2}, topical antifungal ciclopirox in two formulations (“lacquer” and “lotion”), or a combination of visible-light-activated MM **1** plus 405 nm light at 87.6 J cm^{-2} and ciclopirox using a Kruskal–Wallis test with Dunn's multiple comparison test in GraphPad Prism 8.0 (San Diego, CA, USA). Further experimental details are provided in the main text. CPX: ciclopirox. ns: non-significant.

Treatments compared	Mean rank diff.	Significant?	Summary	Adjusted P Value
1% DMSO *vs* CPX "Lotion"	13.33	No	ns	>0.9999
1% DMSO *vs* CPX "Lacquer"	13.67	No	ns	0.9794
1% DMSO *vs* MM **1**	28.00	Yes	**	0.0024
1% DMSO *vs* CPX "Lotion" + MM **1**	37.72	Yes	****	<0.0001
1% DMSO *vs* CPX "Lacquer" + MM **1**	42.28	Yes	****	<0.0001
CPX "Lotion" *vs* CPX "Lacquer"	0.3333	No	ns	>0.9999
CPX "Lotion" *vs* MM **1**	14.67	No	ns	0.7188
CPX "Lotion" *vs* CPX "Lotion" + MM **1**	24.39	Yes	*	0.0151
CPX "Lotion" *vs* CPX "Lacquer" + MM **1**	28.94	Yes	**	0.0014
CPX "Lacquer" *vs* MM **1**	14.33	No	ns	0.7983
CPX "Lacquer" *vs* CPX "Lotion" + MM **1**	24.06	Yes	*	0.0177
CPX "Lacquer" *vs* CPX "Lacquer" + MM **1**	28.61	Yes	**	0.0017
MM *vs* CPX "Lotion" + MM **1**	9.722	No	ns	>0.9999
MM *vs* CPX "Lacquer" + MM **1**	14.28	No	ns	0.8122
CPX "Lotion" + MM *vs* CPX "Lacquer" + MM **1**	4.556	No	ns	>0.9999

Supporting Figures

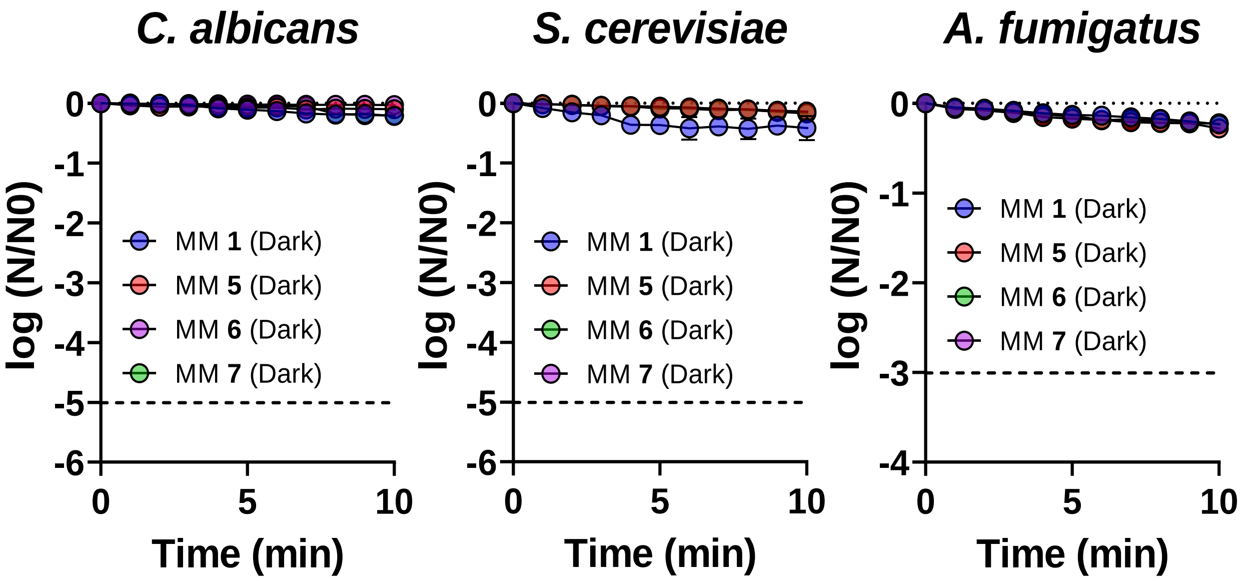

Figure S1. Time-kill curves of different fungal strains treated with 2× MIC of different MMs in the absence of light. The results are the average of at least three independent biological replicates ± the standard error of the mean. Further details on the experimental methodology are provided in the main text.

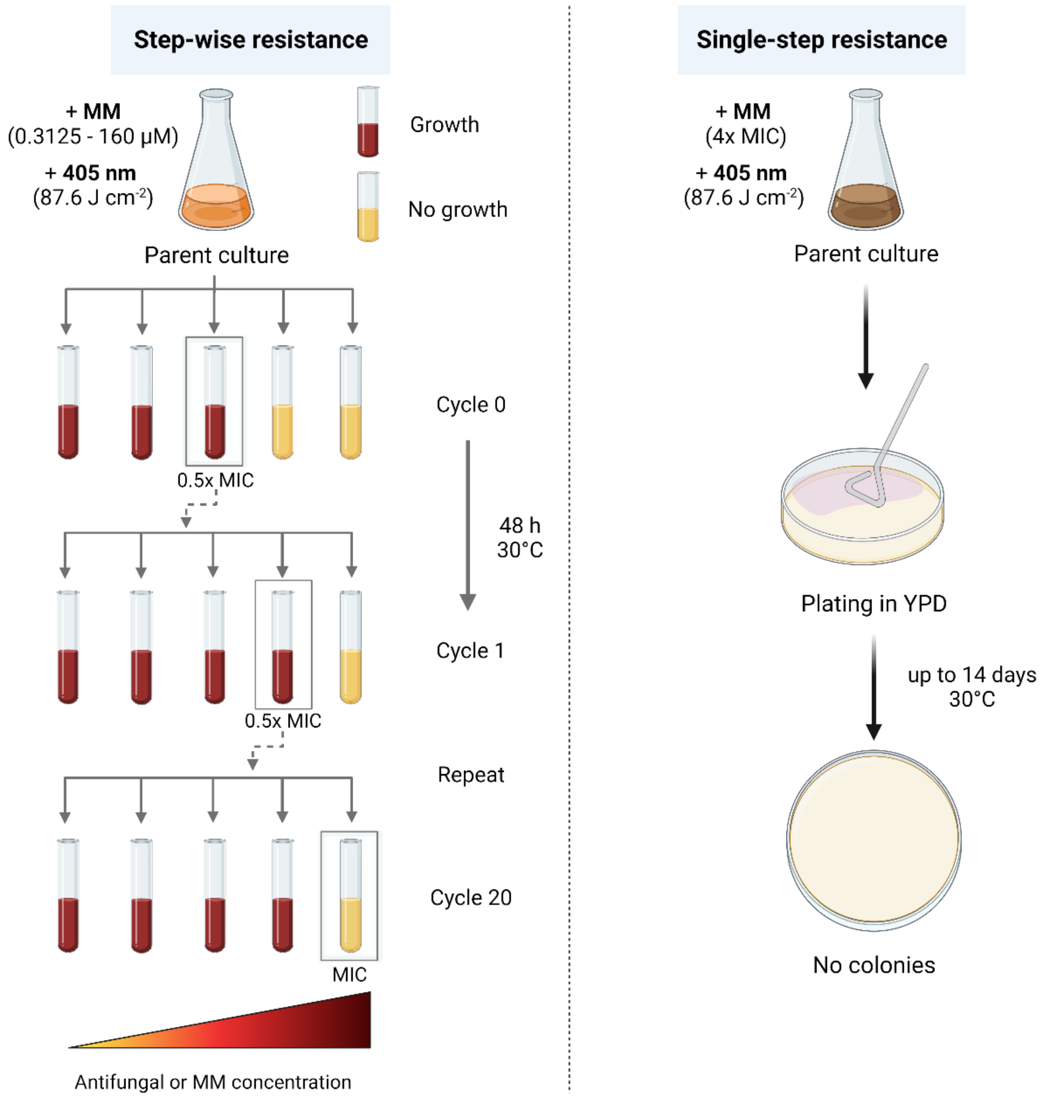

Figure S2. Evaluation of the development of resistance to MMs in *C. albicans*. For the stepwise resistance assessment by serial passage (left), *C. albicans* cell suspensions were treated with increasing concentrations (0.3125–160 µM) of different MMs (8 mM stock in DMSO) and then irradiated with 405 nm light (87.6 J cm^{-2}). The irradiated cell suspensions were then inoculated in MOPS-buffered RPMI 1640 (pH 7.0, Sigma, MO, USA), and the tubes were incubated at 30 °C for 48 h. The minimum inhibitory concentration (MIC) was identified as the concentration of antifungal or MM that resulted in no visible growth after incubation [3]. Cells able to grow at 0.5× MIC of each MM were collected by centrifugation (5,000 × *g*, 5 min), resuspended and re-challenged with a range of MM concentrations and irradiated with 405 nm light (87.6 J cm^{-2}). The procedure was repeated for a total of 20 consecutive cycles. The isolation of MM-resistant mutants was also attempted using a single-step strategy (right) [5], whereby high-density (~10^9 c.f.u. ml^{-1}) cell suspensions of *C. albicans* cells were treated with 4× MIC of the various MMs and irradiated with 405 nm light (87.6 J cm^{-2}). Irradiated cells were then inoculated in YPD and incubated at 30 °C. However, MM-resistant colonies could not be recovered even after 14 days of incubation. Created with Biorender.com.

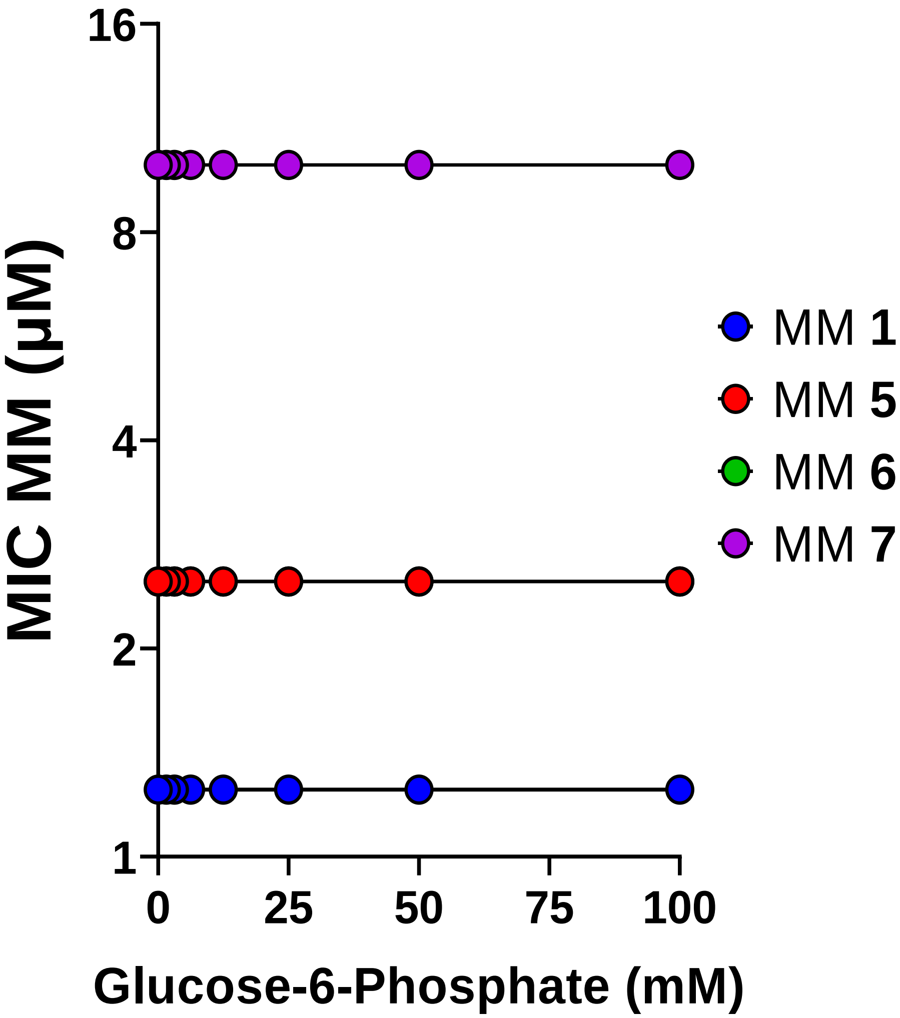

Figure S3. Effect of increasing concentrations of glucose-6-phosphate, used as a representative of the negatively charged polysaccharides of the fungal cell wall, on the MIC of different MMs in *C. albicans* determined by competition binding experiments. Further details on the experimental procedure are provided in the main text. Note that the lines from MM **5** and MM **6** are superimposed. The results are the average of at least three independent biological replicates.

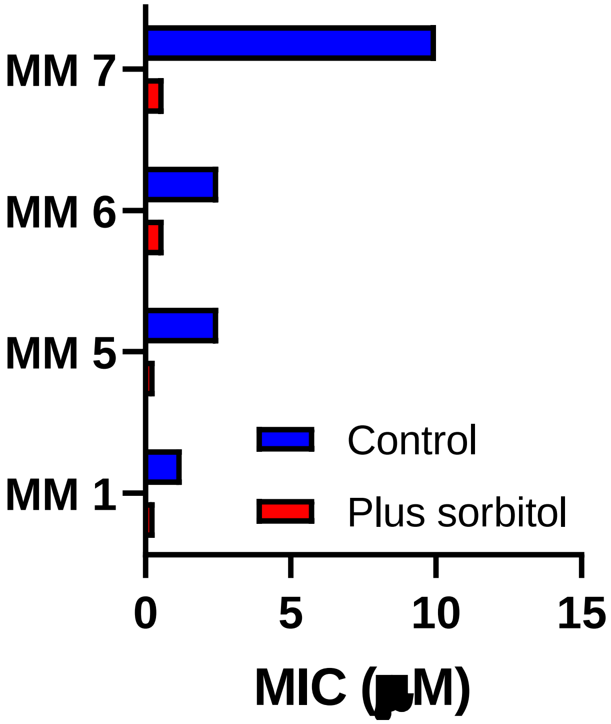

Figure S4. MICs of different visible-light-activated MMs in *C. albicans* grown in the presence and absence of sorbitol (0.8 M). Sorbitol protects cells from drugs that target the fungal cell wall, resulting in an increase in the MIC compared with untreated samples [6]. Experimental details on the determination of the MM MIC can be found in the main text. The results are the average of at least three independent biological replicates.

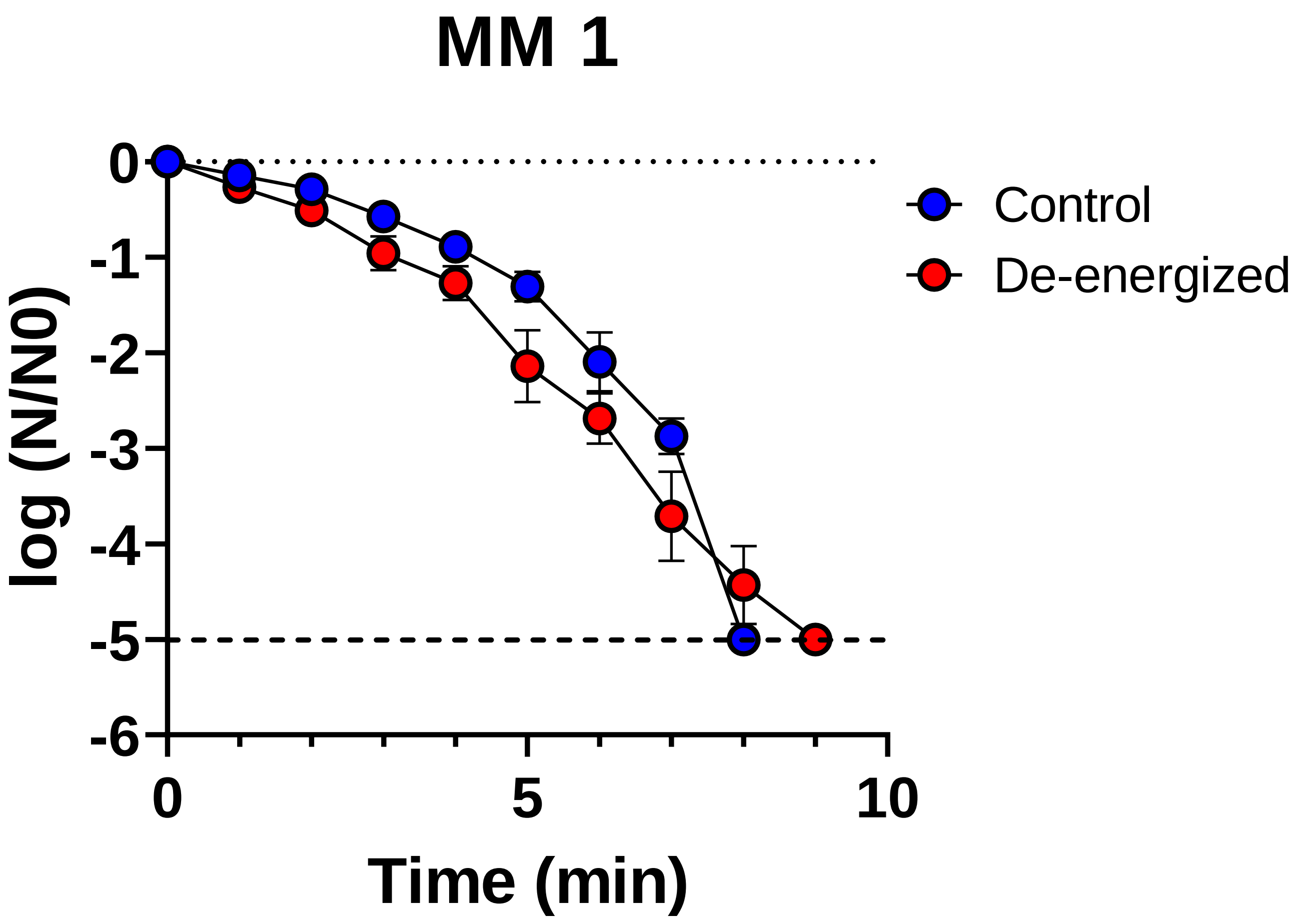

Figure S5. Survival curves of de-energized versus exponential, fully energized cells of *C. albicans* treated with visible-light-activated MM **1** (2× MIC). Energy depletion of *C. albicans* was achieved by resuspending cells in de-energization buffer (1 μM antimycin A, 5 mM 2-deoxy-D-glucose, 50 mM HEPES buffer, pH 7.0) for 3 h, as described for the determination of efflux pump activity in the main text. Survival curves were generated according to the procedure described in the main text for time-kill assays. The dashed line indicates the detection limit of the method. The results are expressed as the average of at least three independent replicates ± the standard error of the mean.

Drug	Target
Rotenone	Complex I inhibitor
Antimycin A	Complex III inhibitor
Sodium azide	Complex IV inhibitor
Oligomycin	ATPase inhibitor

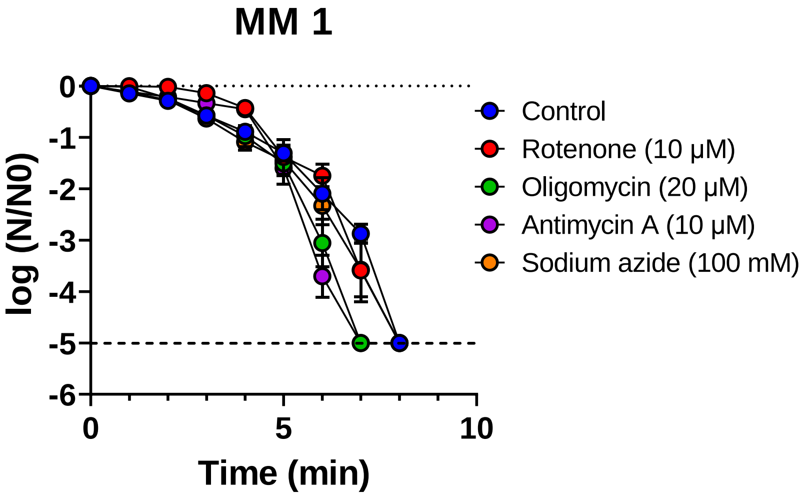

Figure S6. Effect of pre-treatment with drugs targeting different individual components of the electron transport chain (see table inset) on the killing of *C. albicans* by light-activated MM **1** (2× MIC). Survival curves were generated according to the procedure described in the main text for time-kill assays. The dashed line indicates the detection limit of the method. The results are expressed as the average of at least three independent replicates ± the standard error of the mean.

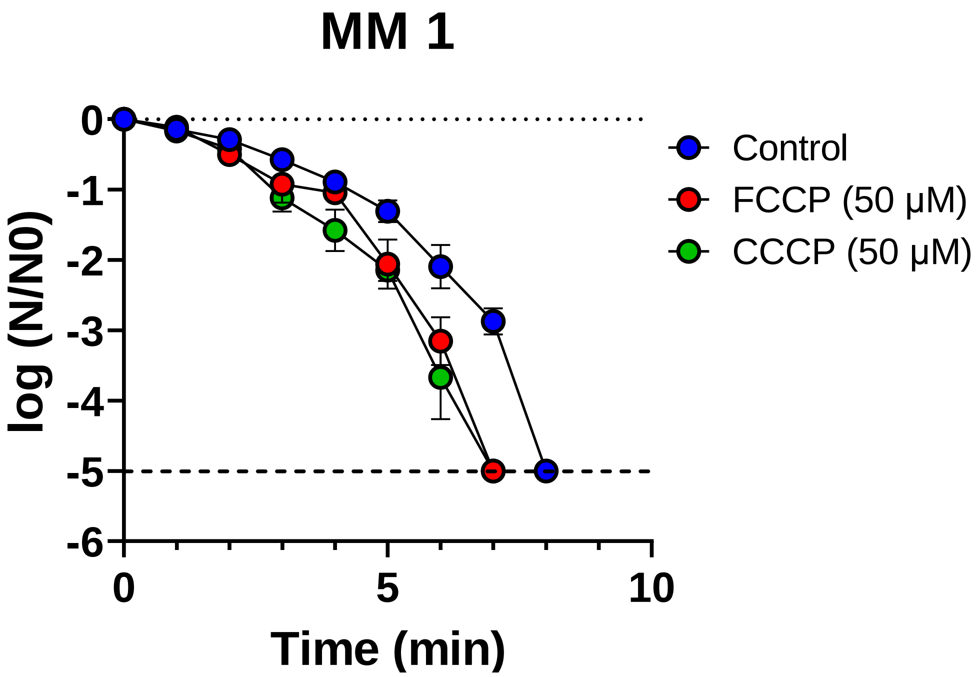

Figure S7. Effect of pre-treatment with the uncoupling agents carbonyl cyanide 4-(trifluoromethoxy)phenylhydrazone (FCCP) and carbonyl cyanide 3-chlorophenylhydrazone (CCCP) on the killing of *C. albicans* by light-activated MM **1** (2× MIC). Survival curves were generated according to the procedure described in the main text for time-kill assays. The dashed line indicates the detection limit of the method. The results are expressed as the average of at least three independent replicates ± the standard error of the mean.

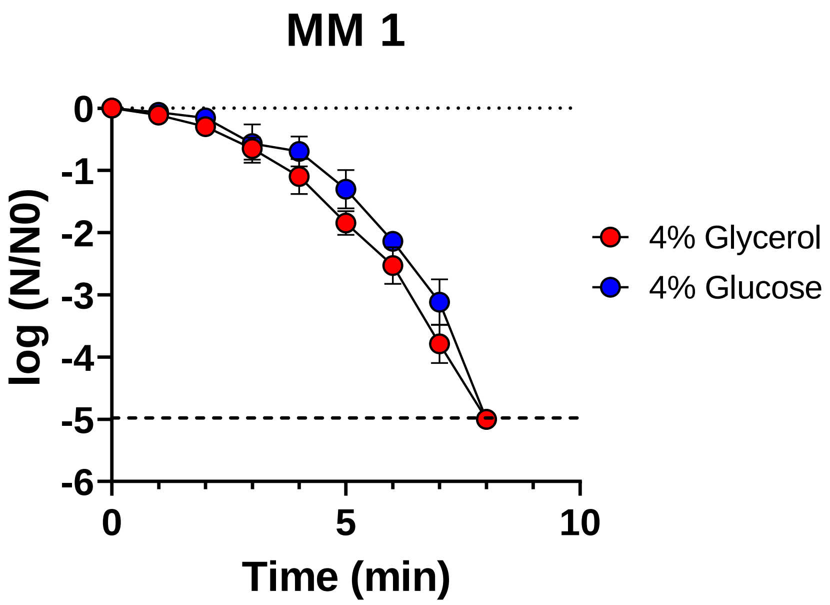

Figure S8. Effect of growth with a fermentable carbon source (glucose) or a non-fermentable carbon source (glycerol) on the killing of *C. albicans* by light-activated MM **1** (2× MIC). Survival curves were generated according to the procedure described in the main text for time-kill assays. The dashed line indicates the detection limit of the method. The results are expressed as the average of at least three independent replicates ± the standard error of the mean.

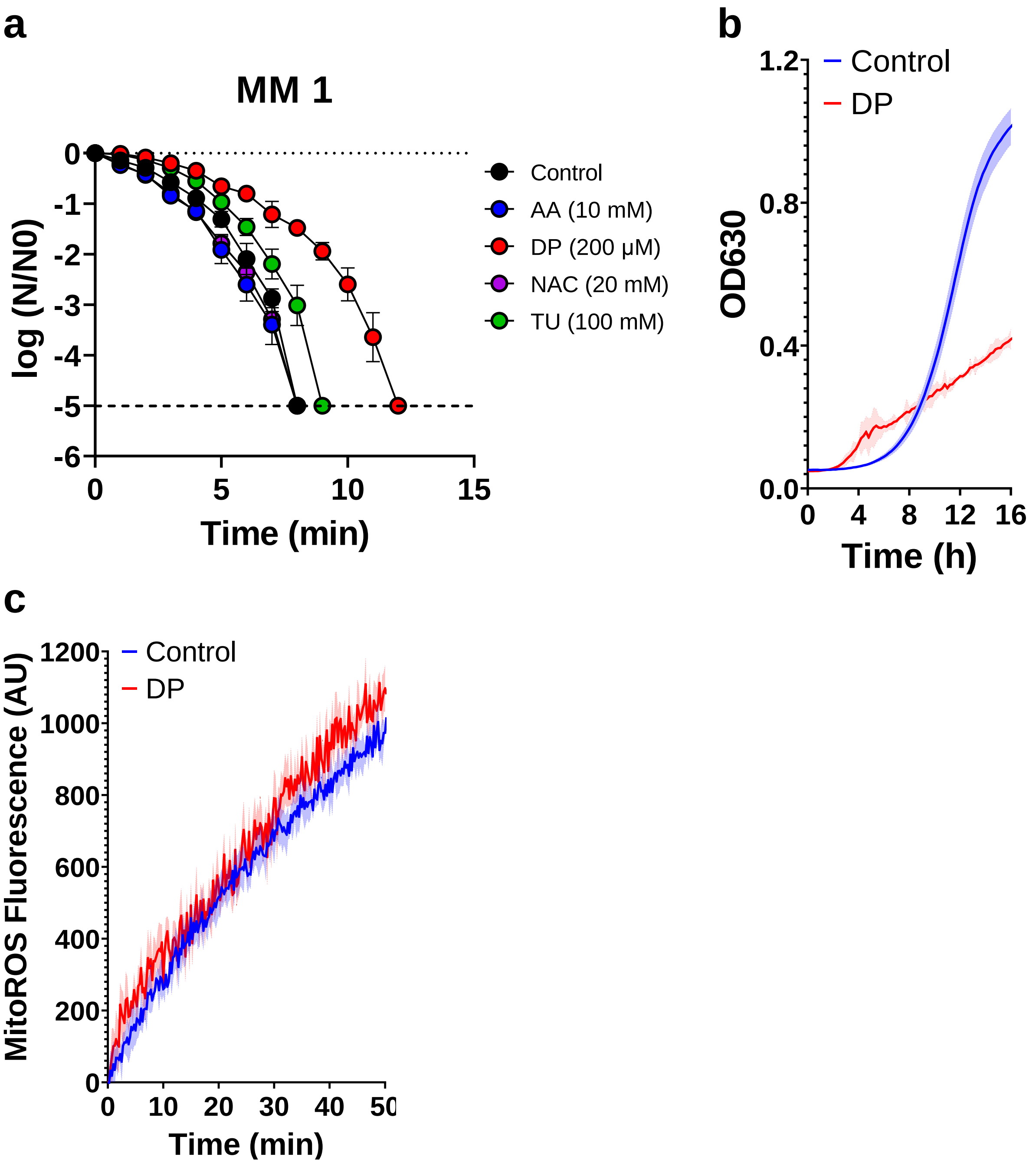

Figure S9. Effect of growth in the presence of different reactive oxygen species (ROS) scavengers and the iron scavenger 2,2'-dipyridyl (DP) on MM **1**-induced killing of *C. albicans*. (**a**) Inactivation profiles of *C. albicans* grown in the presence of different scavengers by visible-light-activated MM **1** (2× MIC). Survival curves were generated according to the procedure described in the main text for time-kill assays. The dashed line indicates the detection limit of the method. The results are expressed as the average of at least three independent replicates ± the standard error of the mean. (**b**) Growth curves of *C. albicans* in the presence and absence of the iron scavenger DP, determined by monitoring the absorbance at 630 nm over time in a microplate reader. The results are shown as the average (line) and standard error of the mean

(shaded area). (**c**) Time profiles of mitochondrial ROS levels in *C. albicans* grown with and without DP after treatment with visible-light-activated MM **1** (2× MIC), detected with the MitoROS^{TM} 580 fluorescent probe, according to the experimental procedure described in the main text. The results are given as the average (line) and standard error of the mean (shaded area). AA: ascorbic acid. DP: 2,2'-dipyridyl. NAC: N-acetyl-cysteine. TU: thiourea.

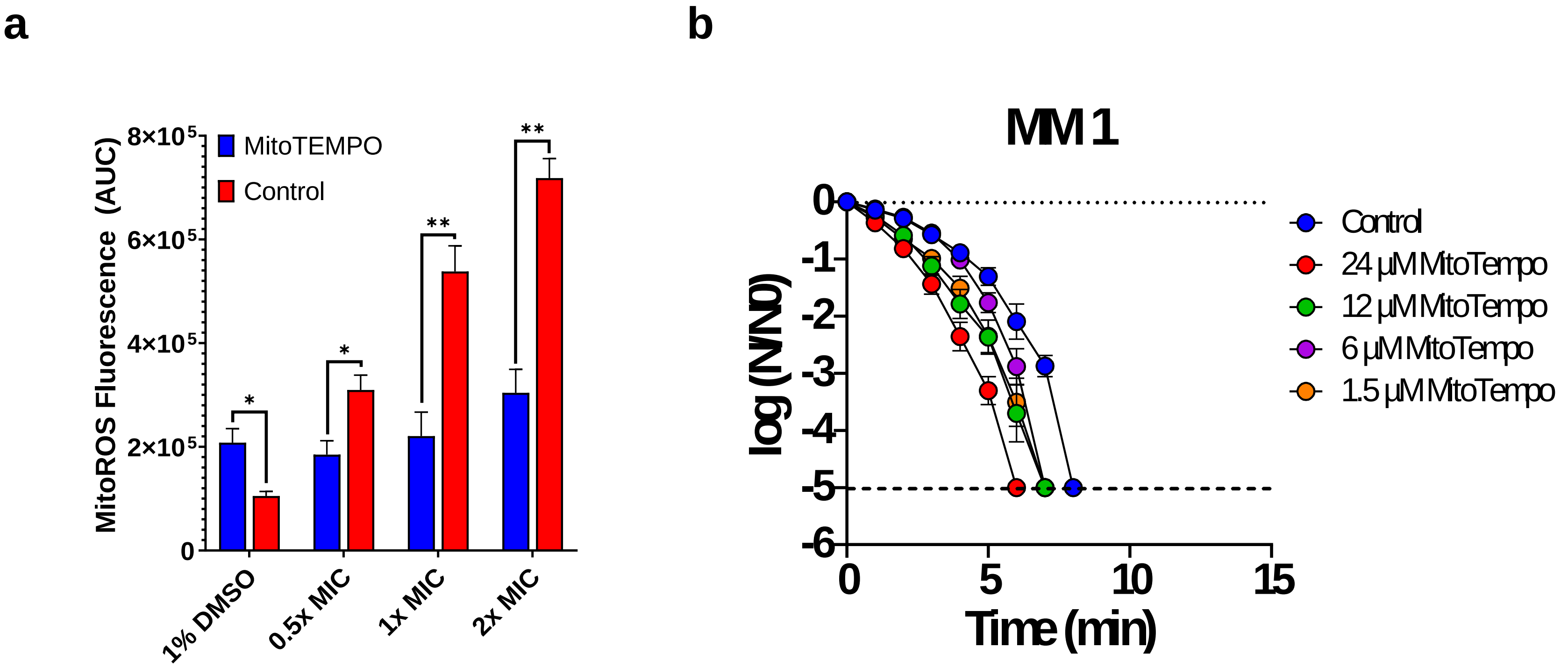

Figure S10. Effect of the mitochondrial superoxide scavenger MitoTEMPO[7] on MM **1**-induced killing of *C. albicans*. (**a**) Mitochondrial ROS levels detected with the MitoROS™ 580 fluorescent probe according to the experimental procedure described in the main text in untreated *C. albicans* cells or cells pre-treated with MitoTEMPO (1.5 µM, MedChem Express, Princeton, NJ, USA), which were then challenged with increasing concentrations of visible-light-activated MM **1**. Asterisks denote the significance of differences in pairwise comparisons performed in GraphPad Prism (San Diego, CA, USA). * $p < 0.05$, ** $p < 0.01$, *** $p < 0.001$, **** $p < 0.0001$. (**b**) Inactivation profiles of *C. albicans* treated with increasing concentrations of MitoTEMPO by visible-light-activated MM **1** (2× MIC). Survival curves were generated according to the procedure described in the main text for time-kill assays. The dashed line indicates the detection limit of the method. The results are expressed as the average of at least three independent replicates ± the standard error of the mean.

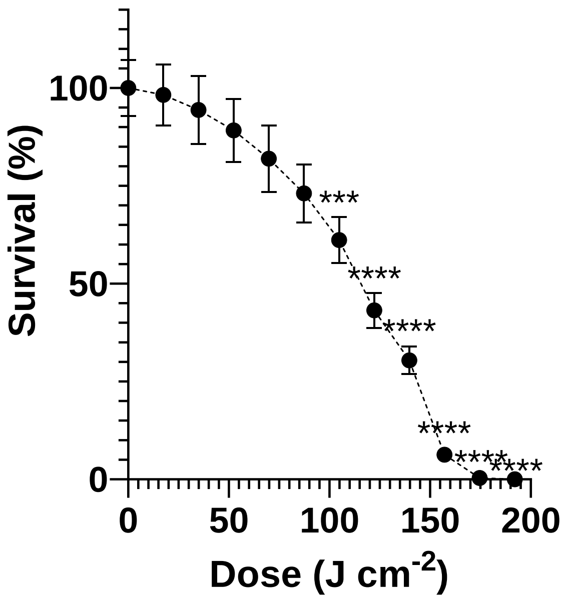

Figure S11. Effect of increasing doses of 405 nm light on the viability of mammalian HEK293T cells. Viability was assessed from ATP levels detected using the CellTiter-Glo® Luminescent Cell Viability Assay. Results are expressed as the average of three biological replicas ± standard error of the mean. Asterisks denote the significance of differences in pairwise comparisons between the viability in unirradiated cells and cells irradiated with different doses of 405 nm light. * $p < 0.05$, ** $p < 0.01$, *** $p < 0.001$, **** $p < 0.0001$. Statistical analysis were performed in GraphPad Prism (San Diego, CA, USA).

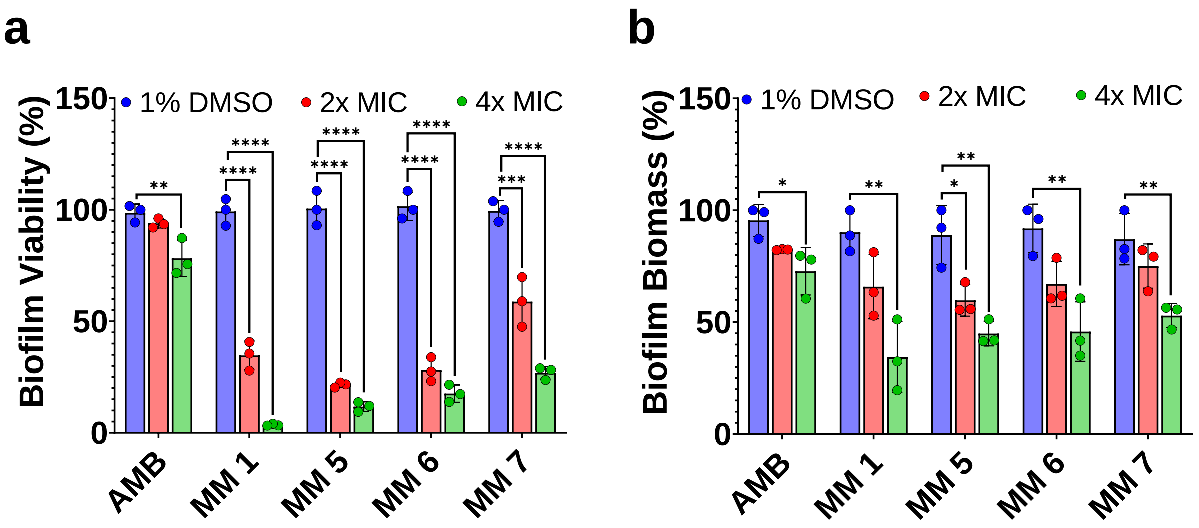

Figure S12. Effects of visible light activated MMs on the biofilms of *Saccharomyces cerevisiae*. (**a**) Reduction of *S. cerevisiae* biofilm viability by amphotericin B (AMB), 1% DMSO or different MMs (2×, 4× MIC) in the presence of 405-nm light (5 min at 292 mW cm^{-2}). (**b**) Reduction of *S. cerevisiae* biofilm biomass by amphotericin B (AMB), 1% DMSO or different MMs (2×, 4× MIC) in the presence of 405-nm light (5 min at 292 mW cm^{-2}). The results are the average of at least three independent replicates ± the standard deviation. Asterisks denote the significance of the differences in pairwise comparisons with 1% DMSO controls performed in GraphPad Prism. * $p < 0.05$, ** $p < 0.01$, *** $p < 0.001$, **** $p < 0.0001$.

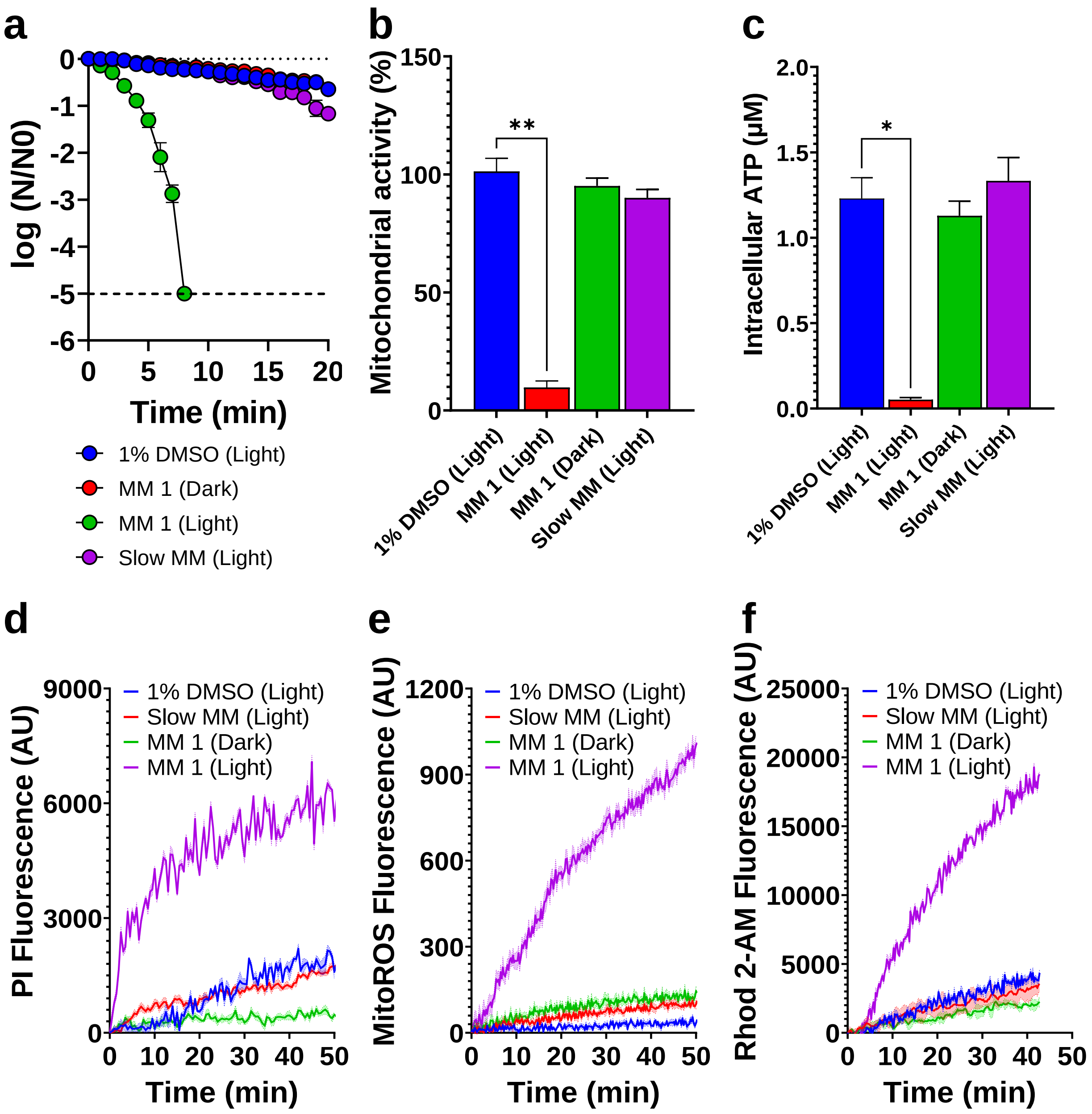

Figure S13. The antifungal activity of MMs against *C. albicans* requires light activation of the fast rotation rates of the motors. (**a**) Time-kill curves of *C. albicans* treated with 1% DMSO, a slow MM (10 μM) or MM **1** (2× MIC) in the presence of 405 nm light (87.6 J cm^{-2}), or MM **1** (2× MIC) in the absence of light activation. (**b**) Mitochondrial dehydrogenase activity in *C. albicans* treated with 1% DMSO, a slow MM (10 μM) or MM **1** (2× MIC) in the presence of 405 nm light (87.6 J cm^{-2}) or MM **1** (2× MIC) in the absence of light activation. (**c**) Intracellular ATP levels in *C. albicans* treated with 1% DMSO, a slow MM (10 μM) or MM **1** (2× MIC) in the presence of 405 nm light (87.6 J cm^{-2}) or MM **1** (2× MIC) in the absence of light activation. (**d**) Temporal profiles of PI fluorescence in *C. albicans* treated with 1% DMSO, a slow MM (10 μM) or MM **1** (2× MIC) in the presence of 405 nm light (87.6 J cm^{-2}) or MM **1** (2× MIC) in the absence of light activation. The lines are the average of at least three biological replicates, and the shaded area is the error. (**e**) Temporal profiles of the MitoROS™ 580 probe used to detect

mitochondrial ROS in *C. albicans* treated with 1% DMSO, a slow MM (10 µM) or MM **1** (2× MIC) in the presence of 405 nm light (87.6 J cm^{-2}) or MM **1** (2× MIC) in the absence of light activation. (**f**) Temporal profiles of Rhod 2-AM used to quantify mitochondrial calcium levels by spectrofluorimetry in *C. albicans* treated with 1% DMSO, a slow MM (10 µM) or MM **1** (2× MIC) in the presence of 405 nm light (87.6 J cm^{-2}) or MM **1** (2× MIC) in the absence of light activation. Asterisks denote the significance of differences in pairwise comparisons performed in GraphPad Prism (San Diego, CA, USA). * $p < 0.05$, ** $p < 0.01$, *** $p < 0.001$, **** $p < 0.0001$. Further experimental details are provided in the main text.

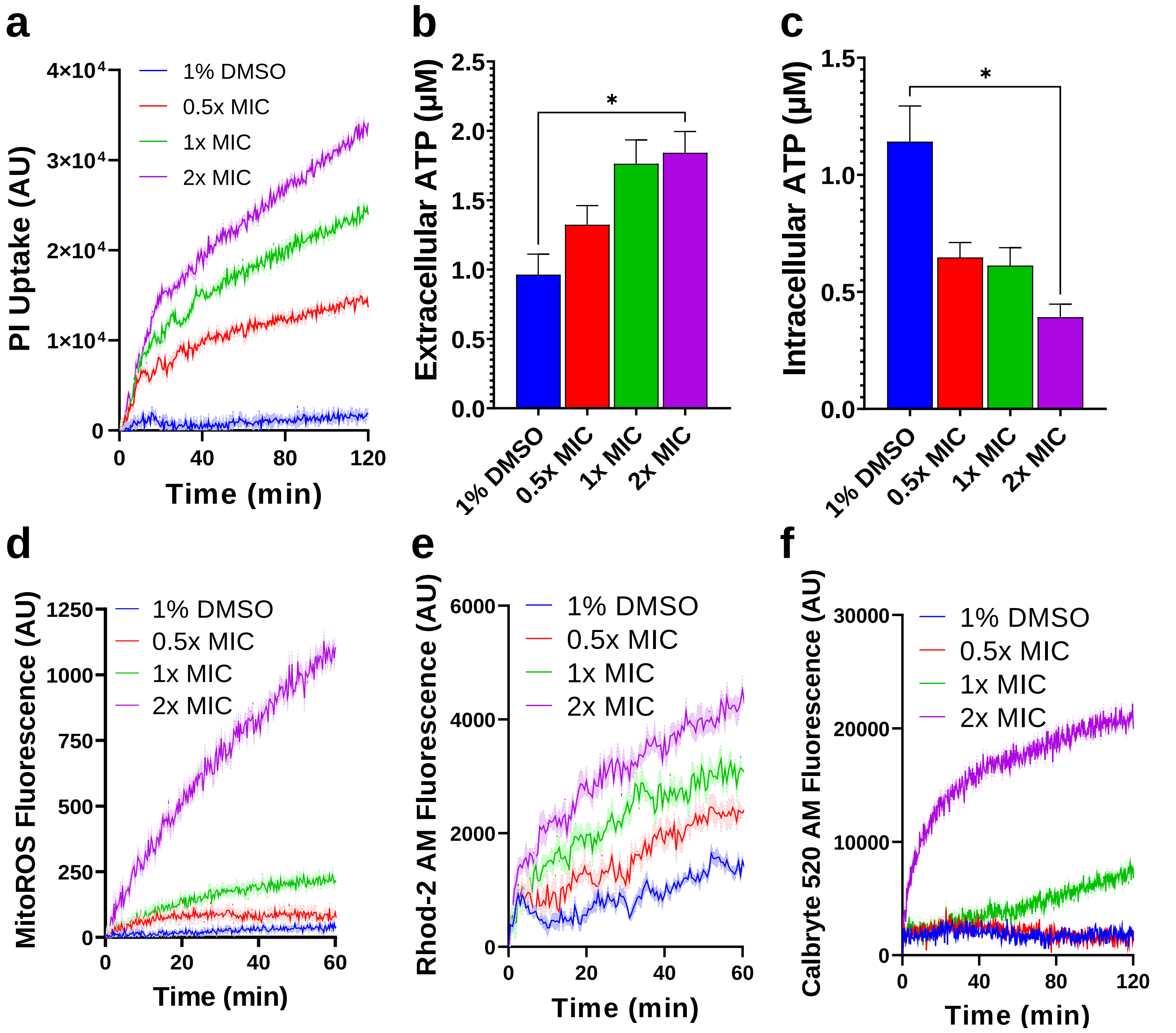

Figure S14. Mechanism of action of visible-light-activated MMs in *Saccharomyces cerevisiae*. (**a**) Representative temporal profile of PI fluorescence after treatment of *S. cerevisiae* with increasing concentrations of MM **1** or 1% DMSO and irradiation with 405 nm light (87.6 J cm^{-2}). The lines are the average of at least three biological replicates, and the shaded area is the error. (**b**) Extracellular ATP levels in *S. cerevisiae* treated with increasing concentrations of MM **1** (0.5–2× MIC) or 1% DMSO and irradiated with 405 nm light (87.6 J cm^{-2}). The results are expressed as the average of at least three independent replicates ± the standard error of the mean. (**c**) Intracellular ATP levels in *S. cerevisiae* treated with increasing concentrations of MM **1** (0.5–2× MIC) or 1% DMSO in the presence of 405 nm light (87.6 J cm^{-2}). Asterisks denote the significance of differences in pairwise comparisons performed in GraphPad Prism (San Diego,

CA, USA). * $p < 0.05$, ** $p < 0.01$, *** $p < 0.001$, **** $p < 0.0001$. (**d**) Temporal profiles of MitoROS^{TM} 580 fluorescence measured by spectrofluorimetry in *S. cerevisiae* treated with increasing concentrations of MM **1** (0.5–2× MIC) or 1% DMSO in the presence of 405 nm light (87.6 J cm^{-2}). Temporal profiles of Rhod-2 AM (**e**) and Calbryte^{TM} 520 AM (**f**) fluorescence obtained by spectrofluorimetry to quantify mitochondrial and cytosolic calcium levels, respectively, in *S. cerevisiae* treated with increasing concentrations of MM **1** (0.5–2× MIC) or 1% DMSO in the presence of 405 nm light (87.6 J cm^{-2}). Lines are the average of at least three biological replicates, and the shaded area is the error.

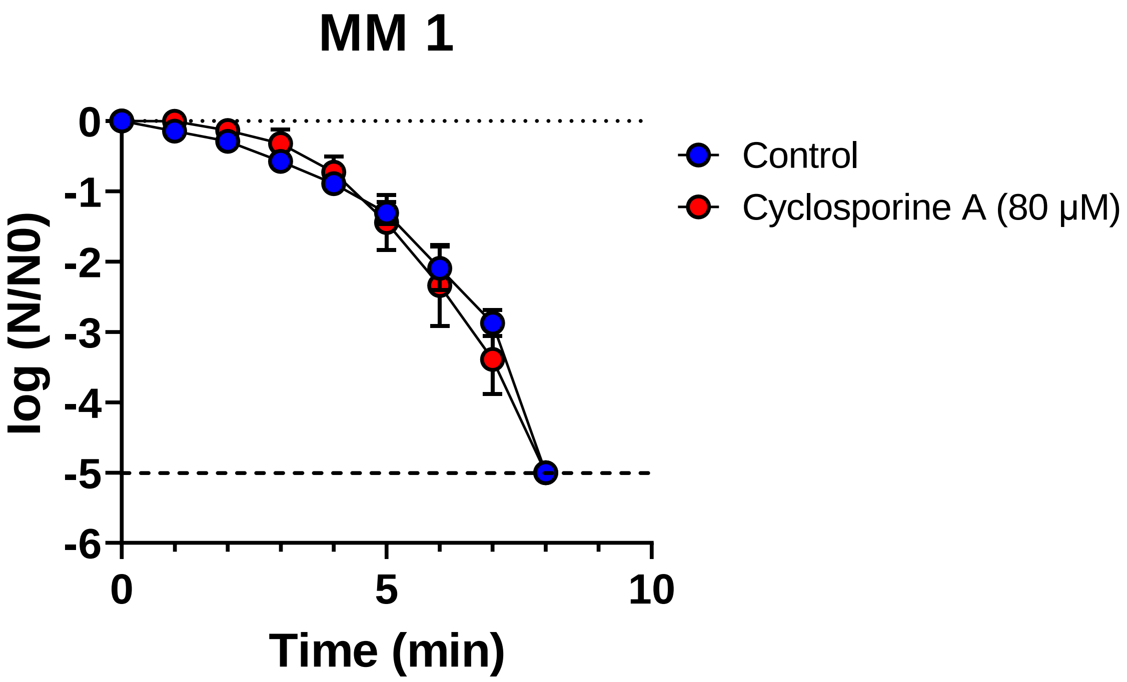

Figure S15. Effect of pre-treatment of *C. albicans* with the calcineurin inhibitor cyclosporin A (80 µM) on susceptibility to killing by visible-light-activated MM **1** (2× MIC). Survival curves were generated according to the procedure described in the main text for time-kill assays. The dashed line indicates the detection limit of the method. The results are expressed as the average of at least three independent replicates ± the standard error of the mean.

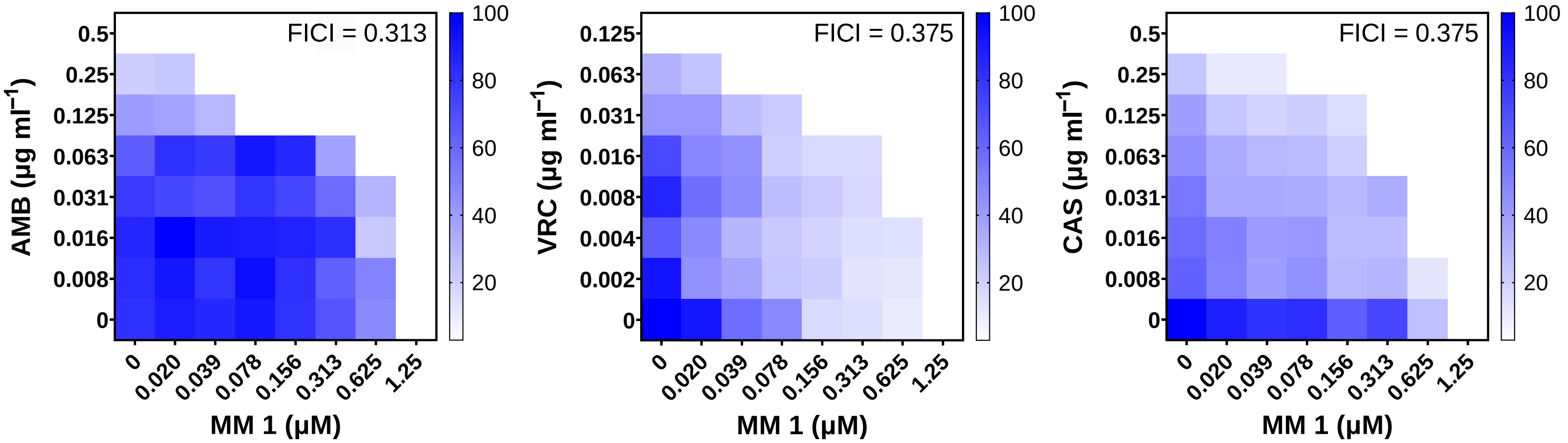

Figure S16. Representative checkerboard patterns showing the interaction between visible-light-activated MM **1** and various conventional antifungal drugs in *S. cerevisiae* and the respective fractional inhibitory concentration indices (FICI) for the interactionS. The results are shown as a heatmap, with the white color denoting no growth (0%) and the blue color denoting growth (100%). Results are the average of three independent replicates. Growth was assessed as the absorbance at 630 nm. AMB: Amphotericin B. VRC: Voriconazole. CAS: Caspofungin.

1

Supplementary Materials and Methods

Synthesis of MM 7

MM 7

GL-26

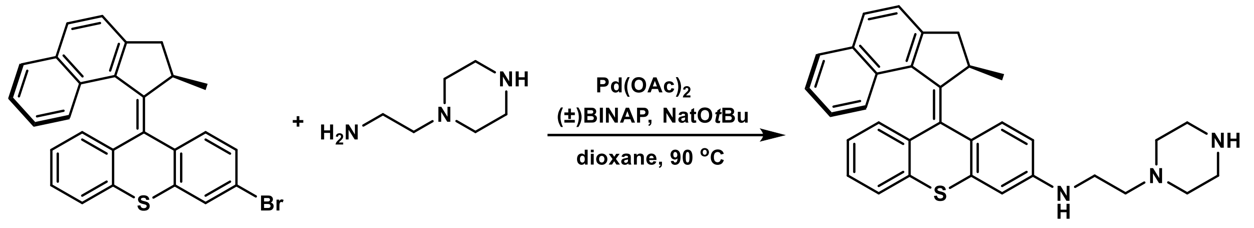

Scheme S1. Synthesis of MM **7**.

All glassware was oven-dried overnight prior to use. Reagent grade dichloromethane (DCM, CH_2Cl_2) was distilled from calcium hydride (CaH_2) under a N_2 atmosphere. All reactions were carried out under a N_2 atmosphere unless otherwise noted. All other chemicals were purchased from commercial suppliers and used without further purification.

GL-26 was synthesized according to previous literature [8]. (±)2,2'-Bis(diphenylphosphino)-1,1′-binaphthyl (BINAP) (15 mg, 0.024 mmol) and palladium(II) acetate (1.8 mg, 0.008 mmol) were mixed in dry dioxane (4.5 ml) in an 8 ml vial. The solution was stirred for 20 min at room temperature. Afterward, NaO*t*Bu (96 mg, 1 mmol), bromo-substituted motor **GL-26** (91 mg, 0.2 mmol) and 2-(piperazin-1-yl)ethan-1-amine (129 mg, 1 mmol) were added. The mixture was stirred at 90 °C overnight. Subsequently, the reaction mixture was cooled to room temperature and treated with H_2O (10 ml) and DCM (10 ml). The organic phase was separated with a separation funnel. The aqueous phase was extracted with DCM (3 × 10 ml), and the organic phases were combined and washed with H_2O (2 × 10 ml). After the volatiles were removed by rotary evaporation, the crude product was purified by column chromatography (silica gel, MeOH: DCM = 10 : 90) to obtain **MM 7** (9-(2-methyl-2,3-dihydro-1H-cyclopenta[a]naphthalen-1-ylidene)-N-(2-(piperazin-1-yl)ethyl)-9H-thioxanthen-3-amine) as a yellow solid product (yield: 90%, Z: E = 5 : 5). ^{1}H NMR (500 MHz, CD_2Cl_2) δ 7.82 (dd, J_1 = 7.97, J_2 = 1.43 Hz, 1H), 7.73-7.69 (m, 4H), 7.63-7.55 (m, 4H), 7.45 (dd, J_1 =8.12, J_2 = 2.20 Hz, 2H), 7.36-7.32 (m, 1H), 7.24-7.13 (m, 3H), 7.01-6.98 (m, 1H), 6.89-6.84 (m, 4H), 6.79-6.76 (m, 1H), 6.70-6.68 (m, 1H), 6.65-6.63 (m, 1H), 6.62-6.58 (m, 1H), 6.50 (d, J = 8.27 Hz, 1H), 5.92 (dd, J_1 = 8.33, J2 = 2.38 Hz, 1H), 4.29 (m, 2H), 3.65 (m, 2H), 3.20 (m, 2H), 3.08 (m, 2H), 2.93 (t, 4H), 2.91 (t. 4H), 2.67-2.56 (m, 10H), 0.76 (dd, J_1 = 22.10, J_2 = 6.74 Hz, 6H). ^{13}C NMR (125 MHz, CD_2Cl_2) δ: 147.3, 146.7, 145.7, 145.6, 144.4, 144.1, 141.1, 138.7, 136.4, 136.3, 135.7, 135.5, 135.3, 135.2, 132.9, 132.9, 129.3, 129.0, 128.9, 128.8, 128.6, 128.5, 128.2, 128.1, 127.6, 127.5, 127.3, 127.2, 126.7, 126.5, 126.1, 126.1, 125.9, 125.8, 125.6, 124.4, 124.3, 123.8, 123.8, 123.7, 111.6, 111.6, 110.5, 110.5, 56.8, 56.7, 53.9, 53.8, 53.7, 53.6, 53.4, 53.3, 53.1, 52.9, 45.4, 45.4, 40.0, 39.8, 39.6, 37.8, 37.8, 19.0, 18.9.

HRMS (ESI) for $C_{33}H_{34}N_3S$ [M+H]: 504.2473. Found: 504.2466.

FTIR (KBr, cm^{-1}): 3048, 2952, 2837, 1599, 1581, 1495, 1455, 1435, 1397, 1308, 1263, 1226, 1156, 1136, 1098, 1049, 1030, 946, 809, 783, 735, 713, 648.

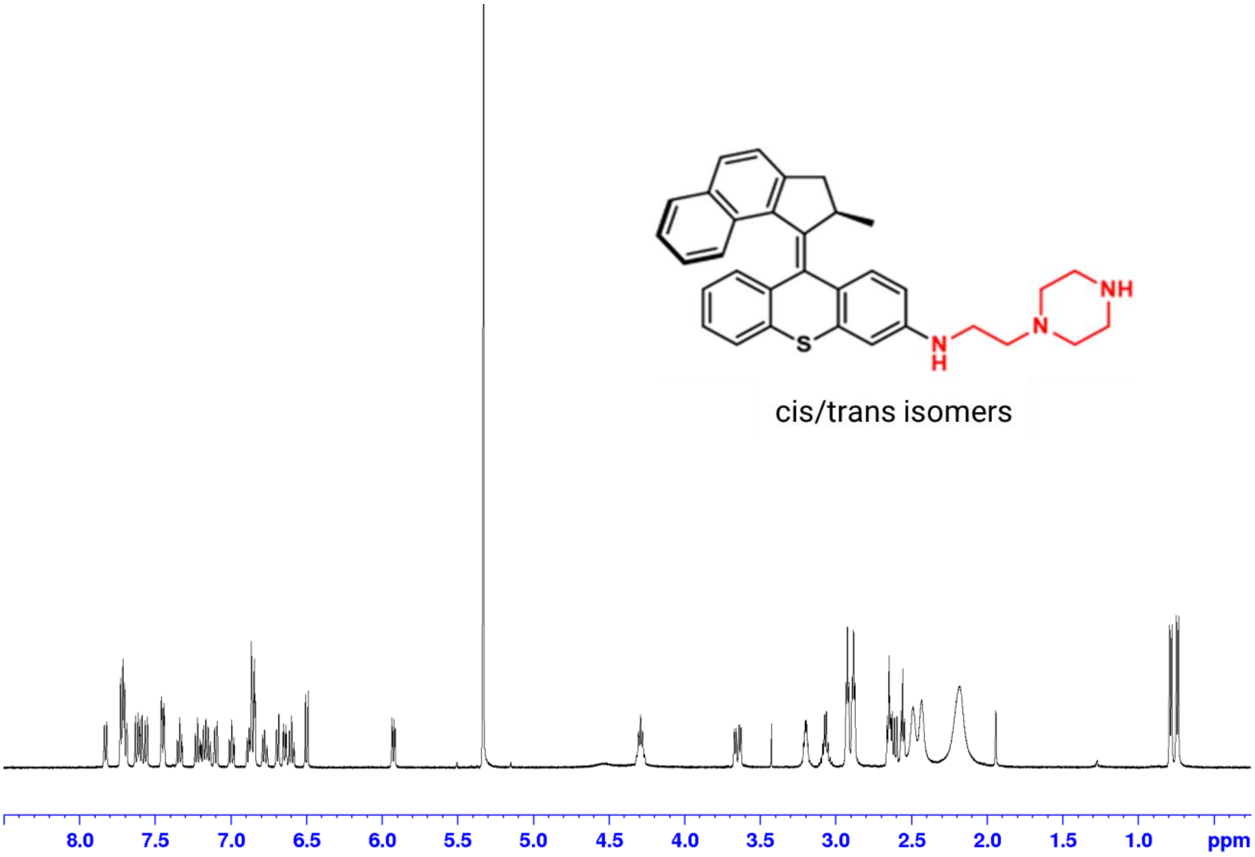

Figure S17. ^{1}H NMR spectrum of MM **7** (GL-82) in CD_2Cl_2

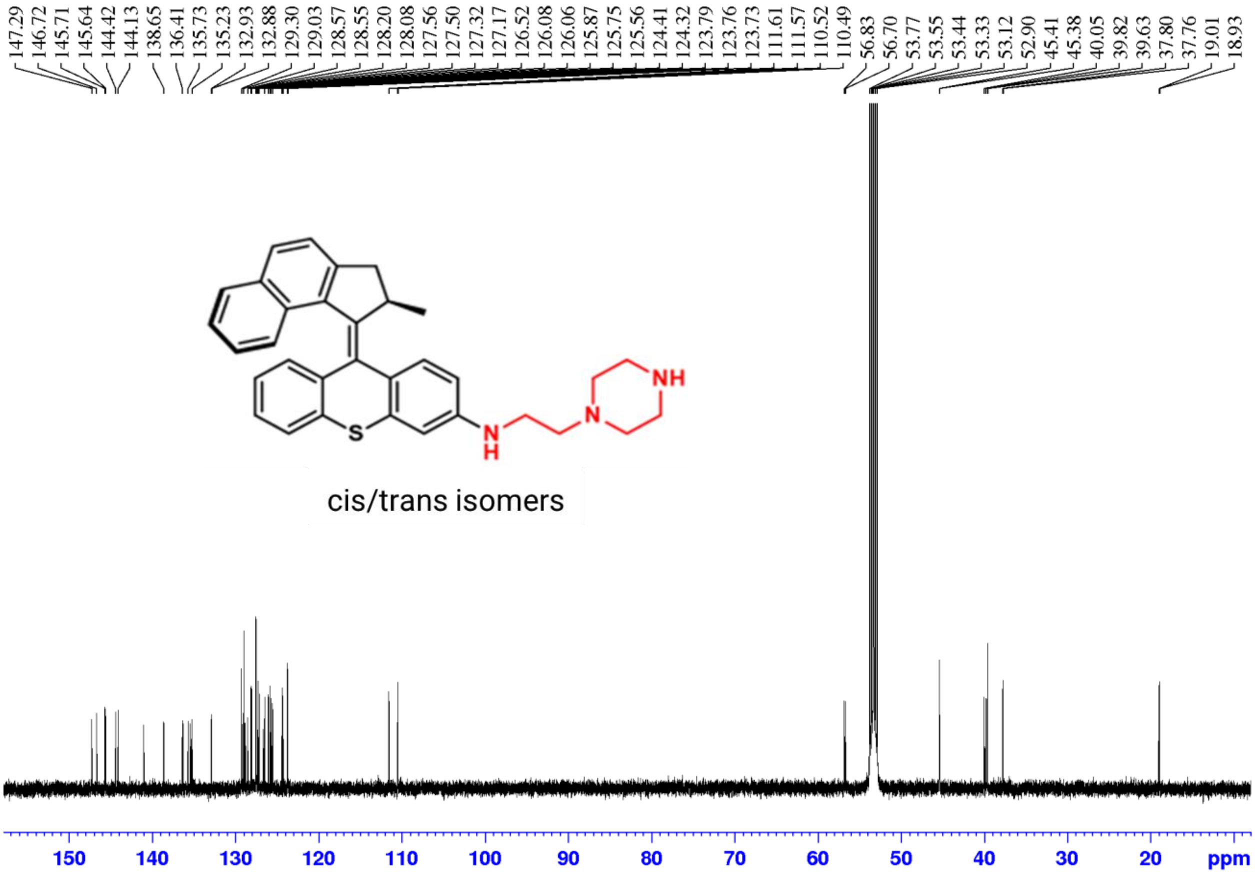

Figure S18. ^{13}C NMR spectrum of MM **7** (GL-82) in CD_2Cl_2

References

[1] M. Klok, N. Boyle, M. T. Pryce, A. Meetsma, W. R. Browne, B. L. Feringa, *J. Am. Chem. Soc.* **2008**, *130*, 10484.

[2] A. L. Santos, D. Liu, A. K. Reed, A. M. Wyderka, A. van Venrooy, J. T. Li, V. D. Li, M. Misiura, O. Samoylova, J. L. Beckham, C. Ayala-Orozco, A. B. Kolomeisky, L. B. Alemany, A. Oliver, G. P. Tegos, J. M. Tour, *Sci. Adv.* **2022**, *8*, eabm2055.

[3] CLSI, *CLSI Doc. M27, 4th Ed.* **2017**.

[4] CLSI, *CLSI Doc. M38-A2* **2008**.

[5] L. B. Rice, R. A. Bonomo, in *Antibiot. Lab. Med. 4th Ed. Williams Wilkins, Balt. Md*, **1996**, pp. 453–501.

[6] D. J. Frost, K. D. Brandt, D. Cugier, R. Goldman, *J. Antibiot. (Tokyo).* **1995**, *48*, 306.

[7] E. J. Helmerhorst, R. F. Troxler, F. G. Oppenheim, *Proc. Natl. Acad. Sci.* **2001**, *98*, 14637.

[8] A. Saywell, A. Bakker, J. Mielke, T. Kumagai, M. Wolf, V. García-López, P.-T. Chiang, J. M. Tour, L. Grill, *ACS Nano* **2016**, *10*, 10945.
